# Supplementary material for: CORAL: A framework for rigorous self-validated data modeling and integrative, reproducible data analysis
Source: Gigascience. 2022 Oct 17;11:giac089. doi: 10.1093/gigascience/giac089 (PMC9575582; doi:10.1093/gigascience/giac089)
Supplement: giac089_GIGA-D-21-00346_Revision_2 [file giac089_giga-d-21-00346_revision_2.pdf]

GigaScience

CORAL: A framework for rigorous self-validated data modeling and integrative, reproducible data analysis

--Manuscript Draft--

**Manuscript Number:**	GIGA-D-21-00346R2
**Full Title:**	CORAL: A framework for rigorous self-validated data modeling and integrative, reproducible data analysis
**Article Type:**	Technical Note
**Funding Information:**	U.S. Department of Energy (DE-AC02-05CH11231) — Not applicable
**Abstract:**	Background<br>Many organizations face challenges in managing and analyzing data, especially when relevant datasets arise from multiple sources and methods. Analyzing heterogeneous datasets and additional derived data requires rigorous tracking of their interrelationships and provenance. This task has long been a Grand Challenge of data science, and has more recently been formalized in the FAIR principles: that all data objects be Findable, Accessible, Interoperable and Reusable, both for machines and for people. Adherence to these principles is necessary for proper stewardship of information, for testing regulatory compliance, for measuring the efficiency of processes, and for facilitating reuse of data analytical frameworks.<br>Findings<br>We present the Contextual Ontology-based Repository Analysis Library (CORAL), a platform that greatly facilitates adherence to all four of the FAIR principles, including the especially difficult challenge of making heterogeneous datasets Interoperable and Reusable across all parts of a large, long-lasting organization. To achieve this, CORAL's data model requires that data generators extensively document the context for all data, and our tools maintain that context throughout the entire analysis pipeline. CORAL also features a web interface for data generators to upload and explore data, and a Jupyter notebook interface for data analysts, both backed by a common API.<br>Conclusions<br>CORAL enables organizations to build FAIR data types on the fly as they are needed, avoiding the expense of bespoke data modeling. CORAL provides a uniquely powerful platform to enable integrative cross-dataset analyses, generating deeper insights than are possible using traditional analysis tools.
**Corresponding Author:**	John-Marc Chandonia<br>Lawrence Berkeley National Laboratory<br>Berkeley, CA UNITED STATES
**Corresponding Author Secondary Information:**	
**Corresponding Author's Institution:**	Lawrence Berkeley National Laboratory
**Corresponding Author's Secondary Institution:**	
**First Author:**	Pavel S Novichkov
**First Author Secondary Information:**	
**Order of Authors:**	Pavel S Novichkov<br>John-Marc Chandonia<br>Adam P Arkin
**Order of Authors Secondary Information:**	
**Response to Reviewers:**	Thank you again for the opportunity to provide this revised manuscript.<br><br>We thank the reviewers for careful review of both the original manuscript and our first revision. All changes relative to our original manuscript were tracked using "track

Powered by Editorial Manager® and ProduXion Manager® from Aries Systems Corporation

changes.” We hope we have addressed all the concerns, as detailed below:

> Reviewer #1: We thank the authors for their efforts to answer the reviewers' questions.

> Outstanding comments:

> 1/. Renaming Results Section to Findings section does not seem possible as this is part of the Giga Science article layout.

This section appears to be appropriate for a Technical Note:

https://academic.oup.com/gigascience/pages/technical_note#Preparing%20main%20manuscript%20text

2/. Cite, in the introduction or general background work about Common Data Elements (CDEs) and ISO11179 specifications, and comment how "contextons" and "microtypes" build on these notions or how they differ.

CDEs are very similar to our dataset-level contextons, and we have added the following text to compare them. The relevant section now reads:

However, a common file format alone is not sufficient to ensure adherence to the FAIR principles of Interoperability and Reusability: in addition to a standard file format, all data, dimensions, and units in these multidimensional arrays must also be formally and rigorously documented. Current state of the art tools such as the CEDAR Workbench [13] greatly facilitate formal documentation of metadata describing entire datasets, but do not readily allow documentation of components such as dimensions and variables. Likewise, Common Data Element (CDE) standards as specified in ISO 111179 [14] have been in use at NIH for over a decade [15], but are not applicable to internal components of data structures.

Later, in discussing dataset-level contextons, we state:

Note that contextons that provide context for the entire matrix are similar to Common Data Elements (CDEs); therefore, microtypes that are compatible with relevant community standards should be included in the set of microtypes defined by the administrators of a CORAL deployment.

Adding rigorous context to all aspects of multidimensional data structures (e.g., the dimension axes of N-dimensional arrays, as well as the data values themselves) is a key aspect of the CORAL data model that was not the focus of CDEs.

3/. REST-API: nice addition to the manuscript. One additional clarifications: is the API compliant with the openAPI/SmartAPI *https://www.openapis.org/* specifications. If the case, it would be another possible point to support FAIR.

OpenAPIs looks like a very useful tool, which we’ll certainly consider using in the next generation of our software, when we re-implement our prototype Python code. As reviewers can see from our current REST API, it’s fairly ad hoc and also only callable by logged in users and authorized remote data access API clients that possess an access token.

A future rewrite of the software that is designed to be accessed by both internal users of an organization as well as the general public (for datasets that have been designated as public) would benefit greatly from a more well-designed and publicly documented API.

4/. Supplementary Material: a lot of material there could have actually be used in the manuscript, eg. figure of the user interface could be used in-lieu of somewhat repetitive

	segments of figures 4-5-6, simply to break the possible monotony of the pictorial work.<br><br>Figures 4, 5, and 6 are essential to understanding the CORAL data model, which we believe is the key innovation in this manuscript. Figure 6 is maybe less critical, and might be moved to the supplementary material if the editors require shortening the main manuscript. We added additional text to the Fig 6 caption to help readers understand when each type of contexton is used:<br><br>For example, the data values in an N-dimensional array are N-dimensional contextons, while the values of variables on each dimension axis are 1-dimensional contextons with the same length as each dimension. Single-value contextons are used to provide context for an entire dataset, and as modifiers in building compound contextons.<br><br>The screenshots of the user interface from our prototype implementation are of more interest to readers interested in the practical aspects of CORAL, so we believe they would be less critical to include in the main manuscript.
Additional Information:	
Question	Response
Are you submitting this manuscript to a special series or article collection?	No
**Experimental design and statistics**<br><br>Full details of the experimental design and statistical methods used should be given in the Methods section, as detailed in our Minimum Standards Reporting Checklist. Information essential to interpreting the data presented should be made available in the figure legends.<br><br>Have you included all the information requested in your manuscript?	Yes
**Resources**<br><br>A description of all resources used, including antibodies, cell lines, animals and software tools, with enough information to allow them to be uniquely identified, should be included in the Methods section. Authors are strongly encouraged to cite Research Resource Identifiers (RRIDs) for antibodies, model organisms and tools, where possible.<br><br>Have you included the information requested as detailed in our Minimum Standards Reporting Checklist?	Yes

**Availability of data and materials**<br><br>All datasets and code on which the conclusions of the paper rely must be either included in your submission or deposited in publicly available repositories (where available and ethically appropriate), referencing such data using a unique identifier in the references and in the "Availability of Data and Materials" section of your manuscript.<br><br>Have you have met the above requirement as detailed in our Minimum Standards Reporting Checklist?	Yes

Powered by Editorial Manager® and ProduXion Manager® from Aries Systems Corporation

Manuscript

Click here to access/download;Manuscript;coral-gigascience-v05.docx

CORAL: A framework for rigorous self-validated data modeling and integrative, reproducible data analysis

Authors: Pavel S. Novichkov[1*], John-Marc Chandonia[1*], Adam P. Arkin[1,2]

* - joint first authors

Corresponding authors:

John-Marc Chandonia

Environmental Genomics and Systems Biology Division

Lawrence Berkeley National Lab

Mailstop Donner

Berkeley, CA 94720-3102

510-292-9495 (phone)

email: JMChandonia@lbl.gov

Adam P. Arkin

Environmental Genomics and Systems Biology Division

Lawrence Berkeley National Lab

Berkeley, CA 94720-3102

email: APArkin@lbl.gov

Affiliations:

1 – Environmental Genomics and Systems Biology Division, Lawrence Berkeley National Laboratory, Berkeley, CA 94720, USA

2 - Department of Bioengineering, University of California, Berkeley, CA 94720, USA

Keywords: FAIR data, Contexton, Microtype, Data Management, Provenance, Data Analysis, Jupyter

Abstract

Background

Many organizations face challenges in managing and analyzing data, especially when relevant datasets arise from multiple sources and methods. Analyzing heterogeneous datasets and additional derived data requires rigorous tracking of their interrelationships and provenance. This task has long been a Grand Challenge of data science, and has more recently been formalized in the FAIR principles: that all data objects be Findable, Accessible, Interoperable and Reusable, both for machines and for people. Adherence to these principles is necessary for proper stewardship of information, for testing regulatory compliance, for measuring the efficiency of processes, and for facilitating reuse of data analytical frameworks.

Findings

We present the Contextual Ontology-based Repository Analysis Library (CORAL), a platform that greatly facilitates adherence to all four of the FAIR principles, including the especially difficult challenge of making heterogeneous datasets Interoperable and Reusable across all parts of a large, long-lasting organization. To achieve this, CORAL's data model requires that data generators extensively document the context for all data, and our tools maintain that context throughout the entire analysis pipeline. CORAL also features a web interface for data generators to upload and explore data, and a Jupyter notebook interface for data analysts, both backed by a common API.

Conclusions

CORAL enables organizations to build FAIR data types on the fly as they are needed, avoiding the expense of bespoke data modeling. CORAL provides a uniquely powerful platform to enable integrative cross-dataset analyses, generating deeper insights than are possible using traditional analysis tools.

One of the Grand Challenges of data science is to facilitate knowledge discovery by enabling datasets to be readily analyzable both by humans and by machine learning algorithms. In 2016, a diverse group of stakeholders formalized a concise and measurable set of principles, called FAIR, to increase the utility of datasets for the purpose of knowledge discovery [1]. The four principles of FAIR are Findability, Accessibility, Interoperability, and Reusability. *Findability* means that data are assigned stable identifiers, and properly indexed. *Accessibility* means the data are easily retrievable by people authorized to have access. *Interoperability* means the data are clearly documented using a formal language, in order to facilitate integrated analyses that span multiple datasets. *Reusability* means the data are documented sufficiently well that datasets may be used by people other than the original data generators, and that the provenance of all data is clear.

The problems of making an organization's data *Findable* and *Accessible* to its members are largely solved by modern databases, including relational databases such as SQL [2] and non-relational "NoSQL" databases such as document stores [3]. The challenges of assigning a unique, permanent ID to each dataset generated within an organization, and then ensuring that the dataset is deposited into a database where it may be retrieved by appropriate people, are largely managerial problems rather than technical ones. On the other hand, making data both *Interoperable* (enabling powerful integrated analyses that span many datasets generated by different teams within an organization) and *Reusable* (enabling later use of datasets by somebody other than the person or team that originally generated the data) is extremely difficult, as we detail below.

The FAIR stakeholders recognized that a number of public databases for specialized data types have formatting standards that mandate rigorous formal documentation of each deposited dataset and its provenance, and that this requirement is sufficient to ensure both *Interoperability* and *Reusability* [1,4]. However, this is not the case for general-purpose datasets, i.e., data types for which rigorously defined formatting and formal documentation standards have not been developed. *Reusability* is challenging for

general-purpose data because non-specialized data storage formats often do not allow or require specification of key details, even basic ones such as units of measurement. As a result, undocumented assumptions and conventions can make it very difficult to reproduce or reuse data. This has produced a reproducibility crisis within the scientific research community [5,6]. Non-reusable data is also very costly: life sciences researchers in the United States alone are estimated to spend over $28 billion each year on irreproducible pre-clinical work [7]. Ensuring *Interoperability* between datasets is challenging for many of the same reasons: when different groups within an organization produce data, impedance matching must be done in order to perform an integrative analysis. Some sources of impedance are differing units, incompatible scaling or normalization of different datasets, and different identifiers used by different groups to refer to the same objects.

The DOE Systems Biology Knowledgebase (KBase) is an open-source software and data platform that facilitates data sharing and integrated analyses of microbial and plant data [8]. KBase defines a large number of data types relevant to such datasets, and each of these data types is formally defined in sufficient detail to ensure compliance with FAIR principles. KBase also provides a Software Development Kit (SDK) that allows users to rapidly import new tools and algorithms into the system. However, the SDK does not currently allow custom data types, and development of new data types is a relatively labor-intensive process that must be done by KBase developers rather than end users [8]. This process of defining new data types is analogous to developing or extending SQL schema: each type must be carefully designed to handle multiple use cases, and must integrate with other data already in the system.

In contrast to KBase, the Galaxy platform [9], also popular among microbial and plant researchers, uses a data model in which files uploaded by users are not validated or parsed in any way. Thus, both Galaxy developers and users can easily introduce new data types into the system to support new tools and algorithms. However, because files in Galaxy are stored without formal type validation - analogous to

documents in a NoSQL database - they are not required to comply with any standards for documentation beyond what is required to successfully run a single tool or algorithm. Thus, the benefit of rapid development in Galaxy comes at the cost of *Interoperability* and *Reusability*. These tradeoffs are illustrated in Figure 1.

In practice, most data types can be represented using a small number of data models, such as arrays, graphs, trees, and hash tables. We surveyed hundreds of data types used by our colleagues in the ENIGMA project, a large consortium of researchers that study how communities of microbes interact with their environment (https://enigma.lbl.gov/). We discovered that the vast majority of data, from raw assays to processed results, can be represented as multi-dimensional arrays of scalars. We believe that this result is generalizable across many fields of research and business, and not just true for ENIGMA. For example, climate modelers widely use the x~~X~~array library for storing data in multi-dimensional arrays, in which key-value pairs are used to label each dimension [10]. Similar libraries for handling labeled multidimensional arrays exist in most computer languages, and file formats such as HDF5 [11] and NetCDF-4 [12] are well supported, mature technologies. However, a common file format alone is not sufficient to ensure adherence to the FAIR principles of *Interoperability* and *Reusability*: in addition to a standard file format, all data, dimensions, and units in these multidimensional arrays must also be formally and rigorously documented. Current state of the art tools such as the CEDAR Workbench [13] greatly facilitate formal documentation of metadata describing entire datasets, but do not readily allow documentation of components such as dimensions and variables. Likewise, Common Data Element (CDE) standards as specified in ISO 111179 [14] have been in use at NIH for over a decade [15], but are not applicable to internal components of data structures.

In this manuscript, we describe the Contextual Ontology-based Repository Analysis Library (CORAL), a novel framework for data modeling and analysis, which aims to achieve an optimal balance between the ease of adding new data types and adherence to FAIR principles.

~~Results~~Findings

An overview of the CORAL data model is shown in Figure 2, and further details are provided in subsequent sections. Our approach to modeling data enables new complex data types to be defined on the fly by users, thus avoiding high maintenance costs, but also ensures that such data types are documented in the formal and rigorous manner that is necessary for *Interoperability* and *Reusability* of all data.

Microtypes and Self-validated Contextons

Formal documentation of data means that all data types are described using a limited vocabulary, in which all terms are clearly defined [1]. To ensure that users document all data in a formal way, CORAL data types must be built using a predefined set of building blocks, which we call *microtypes*.

Microtypes are atomic data types representing a simple concept relevant to a domain of interest (e.g., a gene name, an experimental parameter such as carbon source, or measurements such as optical density or pH). In contrast to complex data types, each microtype should correspond to a conceptual element that can be represented in a single scalar variable. Microtype definitions comprise a unique name, a formal definition of the data type, custom validators, allowed scalar types, and (if applicable) links to other data types. A well-chosen set of microtypes gives users the freedom to model all data types relevant to a domain of interest, and limits ambiguity by restricting the ways in which context can be described to this defined set.

While each microtype defines a concept, additional information is needed every time the microtype is instantiated. In addition to the value of the scalar type, the *units* in which the value is defined provide another piece of critical context. We call this trio (the microtype, its value, and the unit of measurement) a *contexton*, or an atomic unit of context. One or more contextons are required to provide formal context to all data in CORAL. Because the microtype on which a contexton is based includes a validator and a limited set of possible units of measurement, contextons are *self-validating*; i.e., the validity of all data in contextons may be confirmed independently of the contexton's role within larger data structures.

To provide a formal definition of all parts of a contexton, we make extensive use of controlled vocabularies of *ontological terms* [16,17]. Requiring a controlled vocabulary has several advantages: synonymous concepts can be collapsed into a single ontological term, and errors due to misspellings are eliminated. Both the microtype names and the units of measurement are required to be ontological terms, rather than free text. We recommend the use of ontological terms rather than text strings as contexton values, where possible. Designers of microtypes should endeavor to use community standard ontologies wherever possible.

The relationship between contextons, microtypes, and ontologies is shown in Figure 3, below.

Static and Dynamic Data Types

The most FAIR data models require data scientists to completely specify detailed schema for all types of data within an organization, and the links between data types, before new data types can be deployed. This process is often slow and expensive to implement, especially as data types are added or modified over time, and is therefore impractical for dynamically evolving systems. In CORAL, one key design

principle was to provide similar *Interoperability* and *Reusability* as the fully specified schema approach, but without these barriers to adding new data types.

We achieve this goal by dividing data types into two classes: *static core* types that are fundamental to an organization's business or science domain, and thus will remain largely unchanged over time, and *dynamic* types that may be rapidly defined as needed. In practice, only a small fraction (~10%) of data types need to be statically defined. These static core types must be defined in advance, with the associated development costs, but as fundamental types they should not need to be redefined significantly as an organization evolves. Dynamic types, which make up the vast majority of data types, are defined by the users of the system on the fly, with much lower development costs.

Static Core Types

Static core types are complex objects that are fundamental to the domain of an organization. Each static type is defined as a set of contextons, as shown in Figure 4.

In this example, we define a static core type, *Well* (i.e., a groundwater well that is a location in which ENIGMA collects field samples) using a number of microtypes. Although a single Well may be represented by a set of scalar contextons, the set of all Wells in a CORAL instance is represented by a set of ~~1D~~ one-dimensional contextons. This is somewhat analogous to a single table in SQL, except that in CORAL the use of contextons allows all data to be self-validated, and relevant units to be specified.

Dynamic Types

Dynamic data types are most often used for data related to the core types, such as measurements taken on core objects. Dynamic types are defined by combining a limited number of simple, mathematical data structures (e.g., matrices, trees, graphs, etc.) with contextons that provide critical context to the data, in

order to allow users to build a large number of rich, complex data types on the fly. Contextons are used to provide formal context to all parts of the mathematical data structure: each axis, the values, and the object as a whole, resulting in a new dynamic data type. Individual instances of dynamic types (i.e., individual datasets) are called *Data Bricks*. An example is shown in Figure 5.

As discussed above, we found that the mathematical data structure most relevant to ENIGMA data is the multidimensional array. Our first implementation of dynamic types is therefore based on mathematical N-dimensional arrays (NDArrays), but the same approach can be applied to support other mathematical types such as trees or graph structures.

Our NDArray data type is designed to model a homogenous N-dimensional matrix of data, meaning that all the data in the matrix have the same scalar type and units of measurement. Contextons are required to provide context in 3 places:

1. Overall context for the data matrix. The mandatory microtypes *Data Category* and *Brick Name* are used to specify the overall type of data (chosen from an ontology of data categories defined by the instance administrators), as well as a unique name for each dataset. Additionally, each data brick is assigned a permanent *Brick ID* when it is stored in the system. Additional contextons that provide context for the data object as a whole (e.g., the instrument used, or the experiment date), are optional. Note that contextons that provide context for the entire matrix are similar to Common Data Elements (CDEs); therefore, microtypes that are compatible with relevant community standards should be included in the set of microtypes defined by the administrators of a CORAL deployment.
2. Context for each dimension in the matrix. Each dimension has a mandatory microtype, *Dimension Type*, to specify what varies along that dimension. Like *Data Category*, dimension types must be chosen from an ontology of allowed types defined by the instance administrators. Each dimension must contain one or more contextons, each of which describes a single variable

that varies along the corresponding dimension. These contextons usually correspond to the independent variables in an experiment. Each contexton contains a 1-D array of values, with one value for every point along the axis of the dimension. All values within a contexton must have the same units; the contexton also includes a single ontological term documenting the units of measurement.

3. Context for the data values. The values themselves are stored as N-dimensional contextons, which specify the microtype (what is being measured) and the units of measurement. To accommodate heterogeneous data measured under the same conditions, our data model allows multiple sets of homogeneous data values within the same NDArray. Although all such datasets must have the same dimensions, each can include data with a different microtype and units of measurement. These contextons usually correspond to the dependent variables in an experiment.

Each dimension in the NDArray must be labeled by at least one contexton. The microtype for this contexton may be the same as the overall *Dimension Type*. To provide a rigorous formal definition of the data object, the contextons that describe dimensions should be restricted by instance administrators to an informative subset of microtypes. Microtypes that link to core types are included in this set. Other microtypes, such as "comment" (a free text string) are not allowed as dimension contextons in the absence of other informative microtypes, because they do not formally document the data.

It is up to the data generator how best to model their data using this system: for example, a 2-D NDArray with two contextons describing each dimension could also be represented as a 4-D NDArray, with one contexton along each dimension. Often, this decision reflects experimental design: if factorial design is used, it usually makes sense for each independent variable to be a single dimension; on the other hand, if a sparse subset of conditions is chosen in the experiment, it may be more convenient to combine several independent variables into one dimension. Null values (representing sparse datasets or missing data) are allowed.

Data Provenance

Dynamic type definition is a powerful tool that introduces freedom and flexibility to the system. However, at the same time, it can result in isolated "data islands" where data are not sufficiently well documented to determine their provenance (i.e, the inputs and methodology used to produce data). A second key design principle of CORAL is all data must be linked in a network that formally annotates the provenance of these data. This is important both for *Findability*, as datasets that are unrelated to other data in the system may be difficult to find, and also because storing the complete provenance of each object is a key aspect of *Reusability*.

We achieve this goal by requiring that all data objects in CORAL must be linked to core types, either through direct references to core objects or to another dynamic object that ultimately links to a core object. Links between static and dynamic data types are created via the reference mechanism implemented in microtypes, since the same microtypes are used to build context in both static and dynamic data. This concept is similar to foreign keys in SQL. All static and dynamic types are linked in a directed graph, in which the edges document the processes by which one data type is derived from another. Thus, the core types form a "backbone" to which all data must be anchored, and the provenance of every object in the system is clear.

In conclusion, the CORAL data model ensures that data meet all four of the FAIR principles. Data are *Findable* by searching for ontological labels as well as links to other data, and *Accessible* because all versions of all data, microtypes, and ontologies are given permanent IDs and stored in an underlying document store. Our focus on baking *Interoperability* and *Reusability* into our data model greatly

facilitates adherence to these principles as well, without the design overhead of prior general-purpose data modeling approaches.

Functionality

In addition to storing data, CORAL's design enables rich functionality to make the system useful for data analysis, visualization, and managerial oversight. Major features are described in the Methods section. They include a user-friendly upload wizard that guides data providers through the process of formally documenting and uploading datasets, without needing to master technical details such as ontological terms; a powerful search engine that can find datasets according to their provenance as well as searching the contents of the dataset; a plotting wizard that allows non-technical users to visualize their datasets using plot types that are relevant to the dimensionality of the dataset; a remote data access API; and a management dashboard that gives an overview of all data in the system and allows users to drill down to explore particular datasets of interest. CORAL also includes a Python API to enable powerful computational analyses of datasets in Jupyter notebooks [18]. This API provides access to the search engine, retrieves individual datasets and their provenance, simplifies merging of linked datasets to perform integrative analyses, and automatically tracks the provenance of datasets as computational operations (e.g., dimension reduction, clustering, and data transformation) are applied to them. Details of these features are provided in the Methods section, and examples of the UI are shown in the Supplementary Information.

Discussion

For most fields of business or research, information typically flows between three groups: from data generators who create data, to data scientists who analyze it and, to results consumers who use the analyses created by the data scientists in order to make decisions. In ENIGMA, data are generated by wet labs, analyzed by computational biologists, and the results are used by project management to plan future experiments. Each of the three groups has its own language - terms that are critical to documenting data in a *Reusable* and *Interoperable* way. Data scientists who perform analyses typically are more concerned about the mathematical content of the data than about preserving the context provided by the data generators, so conventional tools for data analysis (e.g., R, pandas) focus on the numeric data structure and often lose context.

A benefit of the CORAL data model is that it requires that all context be formally documented by data generators, and it also ensures that this context is not lost during data analysis. Lower-level libraries typically used by data scientists make it easy to lose such context, but CORAL ensures that such context is maintained throughout the data analysis pipeline. Maintenance of context throughout the entire information processing pipeline is critical for the end users of the data (results consumers) to make informed and correct decisions. Users in particular fields of business can build custom solutions that maintain relevant context, but CORAL is a drop-in solution for each industry, once the ontologies and microtypes relevant to that field are chosen or created.

Choosing an appropriate set of microtypes to describe all data in a CORAL instance is analogous to choosing a language for that instance, and must be done by instance administrators prior to uploading data. Additional microtypes can be added to an instance as needed, for example if additional types of measurements will be performed. Microtype names as well as other ontological terms are arranged in

hierarchies, with more specific types and terms linked to more general concepts. Well-designed microtypes and ontologies provide a single controlled vocabulary term to describe a synonymous group of concepts, thus limiting ambiguity. Requiring all contextual labels to be built from contextons rather than free text also eliminates the possibility of errors due to misspelling.

The CORAL approach of building complex data types dynamically using a limited number of mathematical data structures labeled with contextons has several additional benefits:

- The number of mathematical data models is small, and thus many tools and libraries can be implemented in a "generic" way, so that each can operate on many data types in the system. For example, tools to analyze or visualize matrix data could operate on all complex data types that are fundamentally matrices. This is an advantage even over the approach of using fully specified schema for each data type, as such data types are heterogeneous and thus require custom tools.
- Any user (not just "power users" such as instance administrators) can add new *ad hoc* dynamic data types to the system. This is possible because the microtypes and ontologies defined by instance administrators act as a common language for each CORAL instance. Much of the design cost (e.g., data validation) is already built in at the microtype level. Users can construct an enormous variety of complex data types from a few simple, mathematical data models and well-defined contextons, analogous to the enormous variety of objects that can be built out of LEGO bricks.
- Complex dynamic data types made using our model are readily understandable to end users, compared to fully specified schema. Once users are familiar with the language (set of microtypes and ontological terms) used on each instance, the limited number of mathematical data structures underlying our complex types makes the data easy to grasp, and thus improves *Reusability*. Furthermore, by allowing users to define *ad hoc* data types as needed, users can store their data in a format that more naturally reflects their experimental design, rather than shoehorning data into rigidly specified pre-defined schema.

In conclusion, CORAL is a framework that organizations can use to rapidly deploy a powerful, integrated database to catalog, store, and analyze all their data. Large organizations typically generate or use hundreds of types of data, each with a different, often customized, format, including slightly differencing variations on same data types (e.g., a time series vs a condition series; or with vs without replicates). The traditional method of building a bespoke system for data management is time-consuming and expensive, requiring a team of data scientists and database experts. CORAL enables an organization to easily create and re-use templates that capture the unique aspects of each of their hundreds of data types, while also clustering conceptually similar types in order to organize the data and make it easy to understand. Together, these features provide a uniquely powerful tool to enable integrative cross-dataset analyses, generating deeper insights than are possible using traditional analysis tools.

Methods

Details of Microtype structure

Each microtype has the following properties:

- Name - (required) name for this microtype; must be an ontological term, which is unique within a particular CORAL instance
- Description - (required) a formal definition for this microtype. It should not include units; those are provided by a separate "unit" part of each contexton.
- Scalar type - (required) microtype values are restricted to a single scalar type. For example, values for a microtype such as "Concentration" could be restricted to being floating point numbers. Values for a microtype such as "Chemical" could be restricted to being ontological terms.

Use of ontological terms rather than strings as a scalar type for values is highly recommended for two reasons. First, as with the ontology of microtype names, collapsing synonyms into a single term eliminates ambiguity. Second, using ontologies as scalar types enables a powerful form of data validation; e.g., the designer of a data type may require that a user populate a field by choosing between a limited set of possible ontological terms.

For scalar types that are numeric, the microtype designer must specify options for valid units of measurement. As described below in "Details of Contextons," units *must* be specified as ontological terms. The microtype designer may also specify one or more parent classes of ontological terms as valid units (e.g., "concentration unit") in order to assert that all child terms (e.g., "mg/ml") in the ontology are valid units for the microtype.

- Validator - (optional) allows designers to limit the allowed values for this microtype. For example, a string could be required to match a particular regular expression, a numeric type could be input to a function to determine its validity (e.g., to restrict values to non-negative numbers), and ontological terms are restricted to particular ontologies or parts of ontological hierarchies. For example, a "chemical" microtype may be defined so that its allowed values are ontological terms from the ChEBI ontology [19].
- Synonyms/aliases - (optional) synonymous terms for this microtype. This facilitates mapping of input data to microtypes, while limiting ambiguity in contextual descriptions. For example, "ASV", "ESV", "Exact Sequence Variant" and "sub-OTU" are all synonyms for the same concept: a single amplicon sequence that was inferred to be present in a sample prior to the introduction of amplification and sequencing errors.

For all aspects of microtypes that are defined as ontological terms (e.g., name, synonyms, values, and units of measurement), microtype designers should endeavor to use community standard ontologies

wherever possible. This will enable *Interoperability* with other advanced data science applications that use ontologies, since these ontologies can be developed by wide communities and used across diverse data systems.

System Microtypes

To set up a new instance of CORAL, system administrators must define a set of microtypes called "system microtypes," which are mandatory parts of data objects. These include the *Data Type*, *Dimension Type*, *Values Type*, and *Unit Type* microtypes. The allowed values of each of these microtypes is a set of ontological terms. These sets of terms are specified by instance administrators, which allows them to specify broad categories of both static and dynamic data types that may be built in each instance of CORAL. This process is analogous to choosing a language for that specific CORAL instance (see Discussion). When a dataset is uploaded, the specific values of each of these microtypes must be chosen by users from among the allowed terms, in order to formally document the dataset.

Details of Contextons

Values for contextons contain either a single scalar or an array of the same type of scalars; valid scalar types may be defined in the corresponding microtype. The dimensionality of the value or array of values depends on what the contexton is providing context for. See Figure 6 for examples.

Units for contextons *must* be specified as an ontological term, to eliminate ambiguity. By default, unit values may be chosen from the terms in the community standard PATO Units ontology[20], although CORAL also allows instance administrators to define custom units that are relevant to their domain of interest, as defined by the *Unit Type* system microtype.

Compound Contextons

In some cases, context is best described through a combination of atomic microtypes. For example, to express the concept "“concentration of nitrate,", ” multiple contextons are combined to build a single *compound contexton*. The primary contexton includes the microtype that indicates the main property being measured (concentration), while any number of additional contextons may be used to modify the first (e.g., indicating that the molecule whose concentration is being measured is nitrate, or that a series represents technical replicates rather than biological replicates). An example is shown in Fig S-5, in the Supplementary Information.

Implementation

Formatted: Heading 2

A complete prototype implementation of CORAL, including documentation and installation instructions, is available at https://github.com/jmchandonia/CORAL. This prototype consists of two parts, a front end written in Angular, and a back end written in Python. Both parts must be installed on a single host, and the front end may then be accessed from other hosts using a web browser. The prototype code repository also includes sample configuration files, microtypes, data, and installation instructions that enable the installation of an example CORAL instance containing a subset of published ENIGMA data.

In the prototype back end, CORAL’s NDArray dynamic types are built on top of the widely used and well-supported open source xarray library [10]. Each NDArray corresponds to a single xarray *Dataset* object, and reserved key-value pairs are used to store additional metadata (e.g., contextons) that are mandatory in CORAL’s NDArray objects. The xarray library stores and loads each of these *Dataset* objects from the local filesystem in NetCDF-4 format [12]; this format implements a subset of the HDF5 data model [11].

In order to implement the FAIR principles of *Findability* and *Accessibility*, CORAL stores all datasets in the document store of the open source, multi-model database ArangoDB. Ontological terms, metadata about dynamic objects, processes, and each type of static object are stored in an individual collection. As mentioned above, the dynamic datasets themselves are stored on disk in NetCDF-4 format, while the ArangoDB collection indexes metadata such as the ontological terms referenced in each dataset. ArangoDB represents all of the provenance (i.e., process) links between all core data objects and dynamic datasets using a graph database. As a multi-model database, ArangoDB allows queries of the graph database and document store simultaneously, simplifying implementation of the CORAL API. Every data object in CORAL is assigned a permanent identifier, and old versions of data will be marked as superseded by newer versions (with a new identifier) rather than deleted. This process ensures that all versions of all datasets in CORAL are retrievable by their ID at any time.

Dynamic Type Validation

As described above, each microtype includes custom validation methods to ensure that it stores only valid data. To maximize *Interoperability* and *Reusability* of our dynamic data objects, CORAL supports two additional types of data validation: upload templates and use templates. These are described below.

Upload Templates simplify the process of importing data into CORAL by specifying a default set of dimensions and other contextual data for a dynamic data type. When uploading data, the user can optionally add to or remove the default data from the template. For example, a template for microbial growth data might specify a default time series as one dimension, and specify that the values in the matrix are, by default, optical density values with dimensionless units. The template also specifies how to handle invalid data in each underlying microtype, or even specify more strict limits on validating these

data. When new datasets are uploaded, they are validated against all the constraints imposed by their underlying ontological terms. For example, if a field is described by an ontological term that implies a numeric value, all such values are checked to ensure they are numeric before the data can be uploaded. Similarly, if a term indicates that a field is a foreign key linking to a core type, the presence of all linked datasets is verified before the new data are accepted. By default, trying to import invalid data results in an error, preventing it from being uploaded. However, the user can choose, via the templates, whether to treat invalid data as missing (i.e., null values) or to adjust outliers to be within an acceptable range. In these cases, the user is warned of the changes when uploading, but the dataset is accepted.

Use Templates are applied when dynamic datasets stored in CORAL are retrieved for use. When writing a function to manipulate dynamic typed objects, the algorithm developer can specify, using a template, which dynamic objects the algorithm accepts. For example, if an algorithm only works on time series data, the algorithm developer can specify that the algorithm only works with datasets that have at least one dimension labeled with the *Time Series* microtype. Use templates are therefore similar to interfaces in object-oriented languages such as Java and C++.

Search

The provenance graph (Fig 2C), which links each dataset in CORAL with the process and input objects used to generate it, enhances both *Findability* and *Reusability* of static and dynamic datasets. Data are more *Findable* because they can be searched for using graph queries that are supported by the underlying database, in addition to any of the context data within the dynamic type itself (both the microtypes and the ontological terms used as values can be searched). These queries can be quite powerful, for example identifying all data objects that ultimately resulted from work performed by a particular person or group.

The graph of samples and core types also provides additional provenance, using formal ontological terms, for each dynamic type, which is a key aspect of enhancing *Reusability*.

Because ontologies and microtypes are both stored in tree-like hierarchies, CORAL enables users to search by ancestral terms as well; e.g., a search for "carbohydrate" will also find all data that refer to "glucose."

Visualization

CORAL includes "wizards" to build powerful interactive visualizations of dynamic datasets. For multi-dimensional data, the user specifies which dimensions are to be graphed on which axes of the plot. CORAL then generates the plot, labeled according to the appropriate microtypes and ontological terms used in each dimension. Examples are shown in Fig S-4, in the Supplementary Information.

Which plots are available depends on the number of dimensions in each dataset, and the scalar types associated with the microtypes that provide context in each dimension. This feature requires the *Use Templates* functionality described above. For example, to visualize data as a 2-D line graph, the dataset must have at least one dimension labeled with a microtype with numeric values, and also have an overall values type labeled with a numeric microtype. The plotly library [21] is used to draw the plots.

If the number of dimensions in a dataset exceeds the number that can be plotted in a given plot type (e.g., a 4-D dataset on a 2-D line graph), the interface requires additional dimensions to be constrained by the user. Each dimension may be plotted as a series, averaged, or a single value from the dimension may be chosen.

Datasets that contain or are linked in the provenance graph to two specific microtypes, *latitude* and *longitude*, may also be plotted on a Google Map. Map pins may be colored according to any variable in the data brick.

Dynamic libraries

In addition to uploading data, CORAL users can perform analyses of the dynamic data types stored within the system, then save the resulting dynamic data objects. In this case, rather than asking the user to provide provenance for the objects, all provenance is tracked automatically. We wrapped a number of popular Python functions for manipulating data, such as slicing, merging, or other mathematical manipulations such as dimensionality reduction. When these functions are run on dynamic datasets, the object's "session provenance" stores all computational manipulations that have been performed on the data since the time it was retrieved from storage, including records of all methods called and the method parameters. Thus, if the user stores processed results in the CORAL data store, this session provenance is also saved, so that the same process can easily be replicated.

Our NDArray dynamic data type is particularly well suited for modeling observational and physical data. We focus on this data type because observational datasets are among the most heterogeneous and complex-structured data that has to be managed. In addition, observational data must be searchable, sliceable and mergeable in such a way as to facilitate statistical and physical modeling which is often a goal of computational data analysis.

Dynamic joins (step-by-step merging)

To improve *Interoperability* of dynamic data objects, we provide tools to easily merge data from other static and dynamic datasets that are linked in the provenance graph.

For example, the dynamic dataset of geochemical data is linked to the Sample core type, and from there to the Location core type. A user wishing to correlate geochemical data with the sampling locations and/or depths could ask CORAL to automatically retrieve and merge all data from the *Sample* core type as additional variables in the dynamic geochemistry dataset. The user could then merge latitude and longitude data into their dataset by using the *Sample* identifiers to map each data point back to the *Location* core type. The resulting larger dataset could be saved as a new dynamic dataset in the CORAL data store, or analyzed further using the wrapped Python libraries.

An example of Dynamic Joins is shown in Fig S-6, in the Supplementary Information.

Authentication and Permissions

CORAL uses the OAuth 2.0 protocol to manage user access. CORAL instance administrators may add or remove access to individual OAuth identities; however, any user with access to a CORAL instance also has access to all the data stored in that instance. Instance administrators may specify whether a user has access to a bulk upload interface (which allows upload of pre-formatted large datasets, eliminating the Excel steps in the upload wizard described below), as well as a list of static data types they are authorized to upload.

Python API

CORAL provides a unified Python API to support many queries and manipulation of data objects. There are several types of CORAL users, each with their own API needs. Data Providers (e.g., bench scientists or clerical workers) need a specialized interface to upload data, supported by API functionality to map strings to ontological terms, perform data validation, and add new objects to the system. Managers need a specialized interface that gives an overview of the system, with the ability to drill down into particular

objects, and this is supported by a powerful search API. Finally, data scientists will manipulate datasets in Jupyter notebooks, and need APIs for data manipulation, wrappers for popular Python tools, as well as powerful search tools.

Because the CORAL API is built using standard Python libraries for numeric analysis, such as pandas, NumPy, and xarray [10], it is simple for data scientists to use the API to export CORAL data in a number of formats, including simple tab-delimited tables to HDF5 [11] and NetCDF-4 [12].

[18]Additional documentation of our Python API is provided in the Supplementary Information.

REST API and Remote Data Access API

The prototype implementation of the CORAL back end provides includes a REST API that provides data to the front end. Authentication of this API is done using OAuth, with information about the logged in user stored in JWT tokens. The same REST API also allows users who are not logged in, but who with possess a pre-shared cryptographic token, to remotely run the search API and retrieve the resulting static and dynamic datasets. This functionality provides both *Findability* and *Accessibility* of all data in CORAL. This feature also allows developers to build specialized websites to display particular types of data that are stored in CORAL, without requiring users to have accounts on the CORAL server. CORAL uses public-key cryptography to validate the tokens, which are generated in pairs with one of the two tokens secured on the server and the other given to developers authorized to access the data. If the developer token is compromised, access may be revoked by removing the server-side token.

Documentation of our REST API, including our Remote Data Access API, is provided in the Supplementary Information.

Upload Wizard

The uploader was designed to allow non-computer-science users (e.g., bench biologists or other data providers) to upload data. We guide them through providing all the necessary context and provenance for each new dataset using a "wizard." When uploading new data, complete provenance must be provided: all input objects, processes, and other data are then connected in the ArangoDB graph database to the newly uploaded object. The wizard is shown in more detail in the Supplementary Information.

The upload wizard starts by asking the user to document all the data variables and dimensions in the data they are uploading. The interface allows the user to select from dropdowns of allowed data types, units, etc. If similar datasets are uploaded multiple times, instance administrators can create an "upload template" for the corresponding data type, which provides default answers to these questions. Once the user specifies the structure of the data, the wizard creates an Excel template for the user to paste data into. Rows and columns in the template are labeled and colored to reduce the possibility of error. After the user uploads the filled-in template, CORAL interactively allows the user to check the data, then validates the data to ensure that all constraints are satisfied. For example, references to static objects must use the correct names of objects already uploaded into the system.

A final step requires the user to specify the process and personnel that generated the data. This allows CORAL to connect the newly uploaded dataset to the appropriate inputs in the provenance graph.

Management Tools

CORAL provides several interfaces that display an overview of all data in the system, allowing users to drill down to view particular data objects. A management dashboard supports queries of particular interest to managers, such as who is producing which datasets, which datasets are most widely used, etc.

A key aspect of the management dashboard is an overview of the provenance graph, showing all datasets in the system. This is the default view that is displayed to all users upon logging in to CORAL. Double-clicking on individual datasets displays them in the search interface. Checkboxes on the left side of the graph allow users to filter the display to show only a subset of the data; e.g., data produced by a particular project, lab, or person.

Data Scientist View

Data scientists access the CORAL API through Jupyter notebooks, running in a shared directory of a server running JupyterHub. Default permissions are set by the organization. ~~In ENIGMA, t~~The default CORAL permission settings allow any user to read others' notebooks, but writing privileges must be explicitly granted (although a user can fork a writable copy of another user's notebooks). Because all notebooks and data are readable by any user with access to an instance of CORAL, every user may easily reproduce others' analyses.

Availability

CORAL is available under the GNU Affero General Public License ("AGPL") or (at the recipient's option) a separate commercial use license. A complete prototype implementation, including documentation and installation instructions, is available at https://github.com/jmchandonia/CORAL

Competing Interests

The authors declare that they have no competing interests.

Acknowledgements

We thank Gavin Price and other DOE Systems Biology Knowledge~~K~~Base (https://kbase.us) ~~KBase~~ team members for helpful discussions.

This material by ENIGMA (Ecosystems and Networks Integrated with Genes and Molecular Assemblies) (https://enigma.lbl.gov), a Science Focus Area Program at Lawrence Berkeley National Laboratory, is based upon work supported by the U.S. Department of Energy, Office of Science, Office of Biological and Environmental Research, under contract number DE-AC02-05CH11231.

References

1. Wilkinson MD, Dumontier M, Aalbersberg IJJ, Appleton G, Axton M, Baak A, et al.. The FAIR Guiding Principles for scientific data management and stewardship. *Sci Data*. 2016; doi: 10.1038/sdata.2016.18.

2. Codd EF. Relational database: a practical foundation for productivity. *Commun ACM*. 1982; doi: 10.1145/358396.358400.

3. Pokorny J. NoSQL databases: a step to database scalability in web environment. *Proc 13th Int Conf Inf Integr Web-Based Appl Serv*. Ho Chi Minh City, Vietnam: Association for Computing Machinery;

4. Griffin PC, Khadake J, LeMay KS, Lewis SE, Orchard S, Pask A, et al.. Best practice data life cycle approaches for the life sciences. *F1000Research*. 2018; doi: 10.12688/f1000research.12344.2.

5. Baker M. 1,500 scientists lift the lid on reproducibility. *Nat News*. 2016; doi: 10.1038/533452a.

6. Nature: Challenges in irreproducible research. https://www.nature.com/collections/prbfkwmwvz (2018).

7. Freedman LP, Cockburn IM, Simcoe TS. The Economics of Reproducibility in Preclinical Research. *PLOS Biol*. 2015; doi: 10.1371/journal.pbio.1002165.

8. Arkin AP, Cottingham RW, Henry CS, Harris NL, Stevens RL, Maslov S, et al.. KBase: The United States Department of Energy Systems Biology Knowledgebase. *Nat Biotechnol*. 2018; doi: 10.1038/nbt.4163.

9. Goecks J, Nekrutenko A, Taylor J, The Galaxy Team. Galaxy: a comprehensive approach for supporting accessible, reproducible, and transparent computational research in the life sciences. *Genome Biol*. 2010; doi: 10.1186/gb-2010-11-8-r86.

10. Hoyer S, Hamman J. xarray: N-D labeled Arrays and Datasets in Python. *J Open Res Softw*. 2017; doi: 10.5334/jors.148.

11. The HDF Group. Hierarchical Data Format, version 5. HDF Group.

12. Unidata: Unidata | NetCDF. Netw. Common Data Form NetCDF Boulder CO UCARUnidata. http://doi.org/10.5065/D6H70CW6 (2020).

13. Gonçalves RS, O'Connor MJ, Martínez-Romero M, Egyedi AL, Willrett D, Graybeal J, et al.. The CEDAR Workbench: An Ontology-Assisted Environment for Authoring Metadata that Describe Scientific Experiments. *Semantic Web--ISWC Int Semantic Web Conf Proc Int Semantic Web Conf*. 2017; doi: 10.1007/978-3-319-68204-4_10.

14. 14:00-17:00: ISO/IEC 11179-3:2013. ISO. https://www.iso.org/cms/render/live/en/sites/isoorg/contents/data/standard/05/03/50340.html Accessed 2022 Jul 11.

15. Grinnon ST, Miller K, Marler JR, Lu Y, Stout A, Odenkirchen J, et al.. National Institute of Neurological Disorders and Stroke Common Data Element Project – approach and methods. *Clin Trials*. SAGE Publications; 2012; doi: 10.1177/1740774512438980.

16. Studer R, Benjamins VR, Fensel D. Knowledge engineering: principles and methods. Data Knowl Eng 25(1-2):161-197. *Data Knowl Eng*. 1998; doi: 10.1016/S0169-023X(97)00056-6.

17. Gruber T. Toward Principles for the Design of Ontologies Used for Knowledge Sharing. *Int J Hum-Comput Stud*. 43:907–281995;

18. Kluyver T, Ragan-Kelley B, Pérez F, Granger B, Bussonnier M, Frederic J, et al.. Jupyter Notebooks – a publishing format for reproducible computational workflows. In: Loizides F, Scmidt B, editors. IOS Press;

19. Hastings J, Owen G, Dekker A, Ennis M, Kale N, Muthukrishnan V, et al.. ChEBI in 2016: Improved services and an expanding collection of metabolites. *Nucleic Acids Res*. 2016; doi: 10.1093/nar/gkv1031.

20. Gkoutos GV, Schofield PN, Hoehndorf R. The Units Ontology: a tool for integrating units of measurement in science. *Database*. 2012; doi: 10.1093/database/bas033.

21. Plotly Technologies Inc.. Collaborative data science. Montréal, QC;

Figure Captions

Figure 1: Tradeoffs between adherence to FAIR principles and the ease with which new data types can be added to a platform. More rigorous formal definitions of data types facilitate higher degrees of *Interoperability* and *Reusability*, but at the cost of a higher degree of difficulty in adding new data types to the system.

Figure 2: A) to rigorously document context for all data, we introduce the concept of a "contexton," or unit of context. Contextons are built using "microtypes," which we define as atomic data types representing a simple concept relevant to a domain of interest. Both rely on ontologies, which define a controlled vocabulary for describing this domain. Together, the microtypes and ontologies defined for a particular instance of CORAL represent a language that allows users to formally describe all data in that instance in a way that is both *Interoperable* and *Reusable*. B) Dynamic data types, which make up the vast majority of data in a CORAL instance, are defined by the users of the system as needed. These types are built by combining commonly used mathematical data structures with contextons. A limited number of static core types, which are fully specified traditional data structures, are also built using contextons in order to ensure *Interoperability* with the dynamic data. These static core types include the system type *Process*, which is a special core type needed to document the provenance of each data object in a CORAL instance. C) All static and dynamic data in a CORAL instance are referenced in an object graph, where nodes are static or dynamic datasets, and edges are processes. This graph formally annotates the provenance of all data.

Figure 3: A microtype is an atomic variable representing a single concept relevant to the field of interest, typically one which can be described using a single scalar value and (where applicable) a unit of

measurement. Both the microtype names and units of measurement are required to be ontological terms. As part of the microtype definition, values must be constrained to be a simple scalar type, ontological term, or a reference to a data object. A contexton is an instantiated microtype, in which the value and units are specified.

Figure 4: Definition of the static core type "Well" - a location in which environmental samples are taken by ENIGMA field researchers. A number of attributes, such as the name, biome, latitude, and longitude, are relevant to describing each well. Well names are free text, but the biome must be chosen from among a collection of biome terms described in the ENVO ontology. Microtypes are used to ensure that each well's latitude and longitude are specified in degrees.

Figure 5: Definition of a dynamic data type to describe the results of a fitness assay. The first dimension specifies the conditions under which researchers measured fitness values of different genes. The second dimension might specify genes, while a third dimension might specify replicates. Because particular fitness datasets may have any of these dimensions, or more, they are more readily modeled as dynamic datasets rather than as static core objects.

Figure 6: Examples of single-value (i.e., scalar), one-dimensional, and N-dimensional contextons. Contextons are of the same dimensionality as the mathematical data object that they provide context for. For example, the data values in an N-dimensional array are N-dimensional contextons, while the values of variables on each dimension axis are 1-dimensional contextons with the same length as each dimension. Single-value contextons are used to provide context for an entire dataset, and as modifiers in building compound contextons.

Click here to access/download;Figure;allfigs-1.pdf

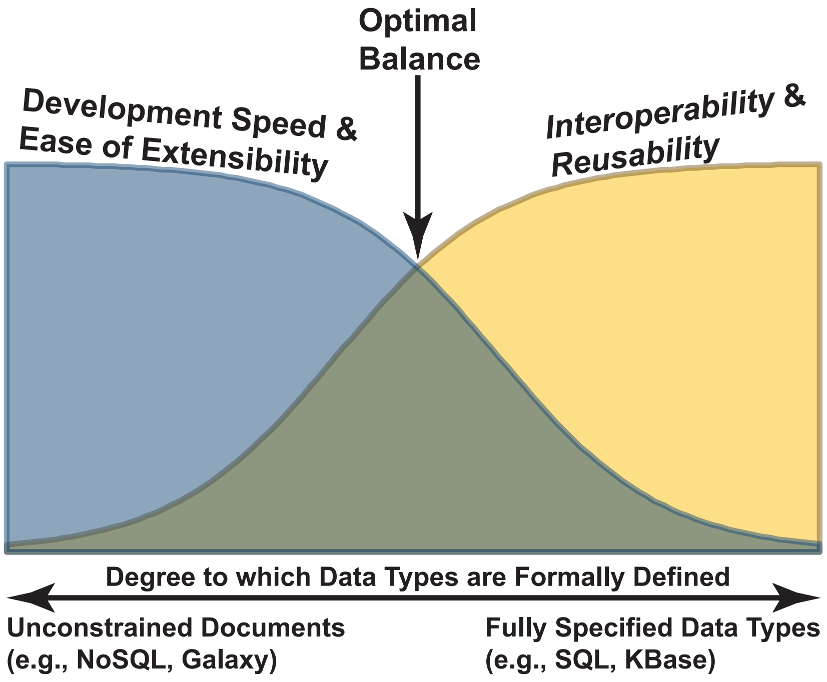

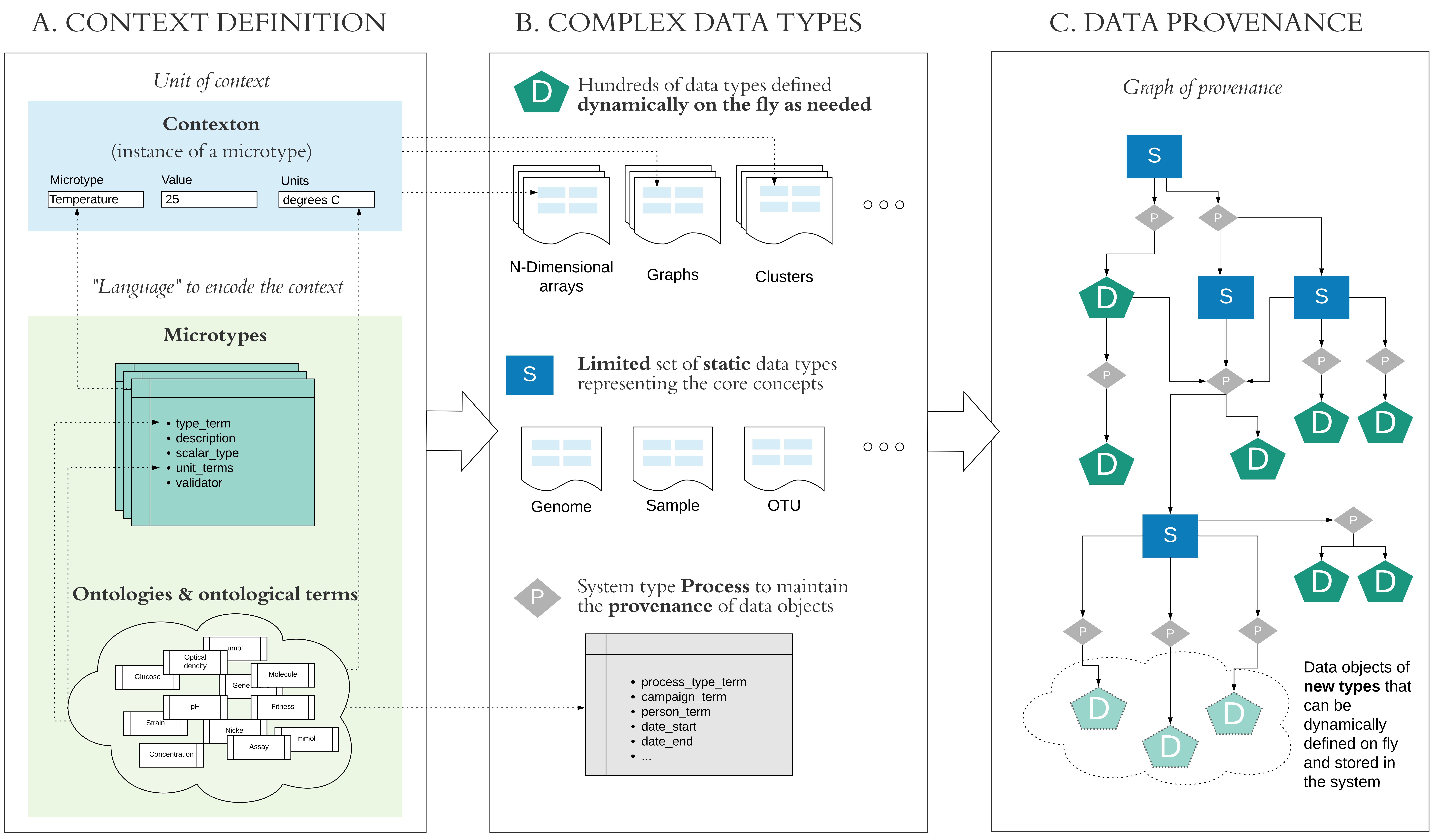
A. CONTEXT DEFINITION
B. COMPLEX DATA TYPES
C. DATA PROVENANCE
Unit of context
Contexton
(instance of a microtype)
Microtype
Value
Units
Temperature
25
degrees C
"Language" to encode the context
Microtypes
• type_term
• description
• scalar_type
• unit_terms
• validator
Ontologies & ontological terms
umol
Optical dencity
Glucose
Molecule
Gene
pH
Fitness
Strain
Nickel
mmol
Assay
Concentration
D
Hundreds of data types defined dynamically on the fly as needed
N-Dimensional arrays
Graphs
Clusters
S
Limited set of static data types representing the core concepts
Genome
Sample
OTU
P
System type Process to maintain the provenance of data objects
• process_type_term
• campaign_term
• person_term
• date_start
• date_end
• ...
Graph of provenance
Data objects of new types that can be dynamically defined on fly and stored in the system

SELF-VALIDATED CONTEXTONS

powered by microtypes

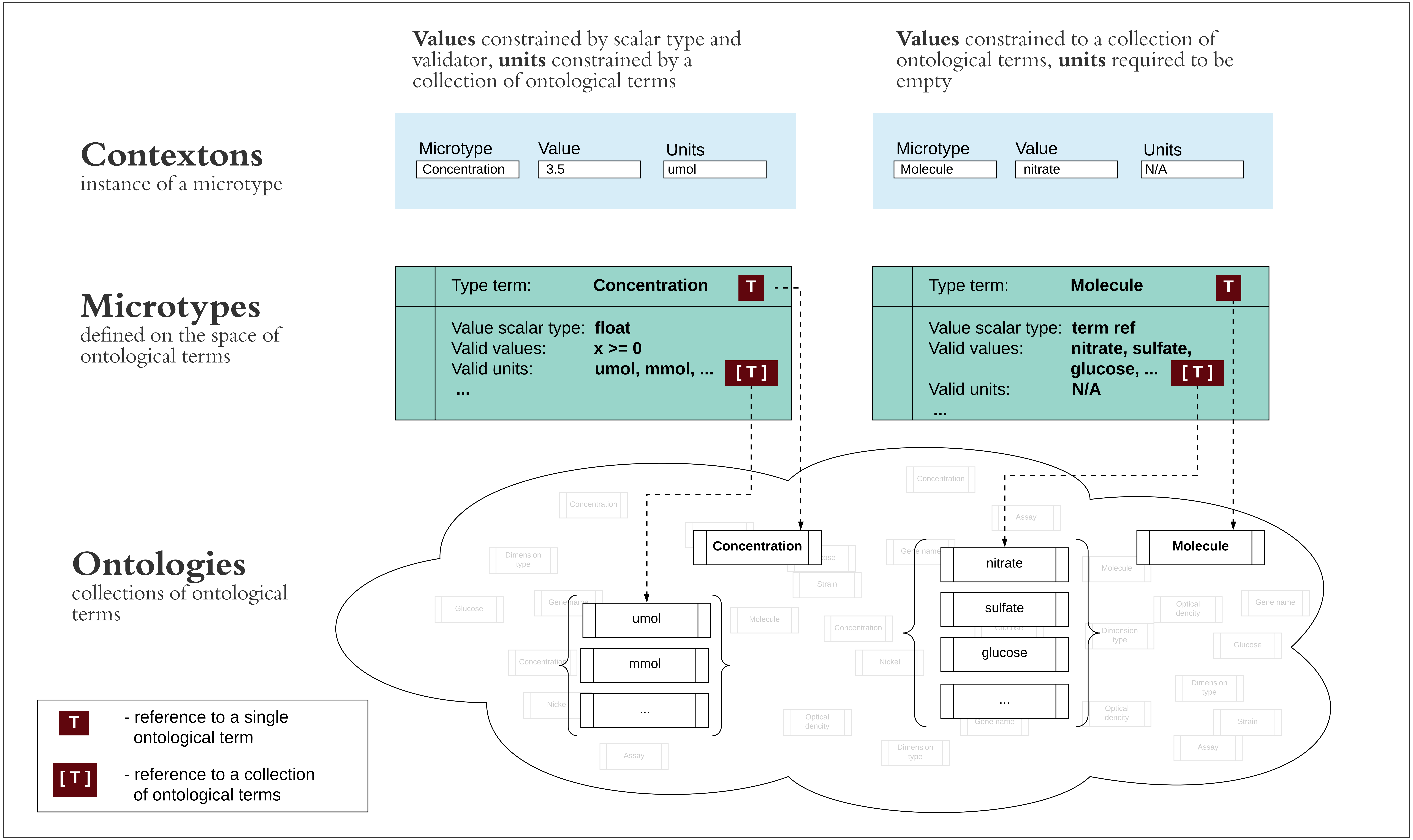

CORE TYPES DEFINED BY 1D CONTEXTONS

powered by microtypes

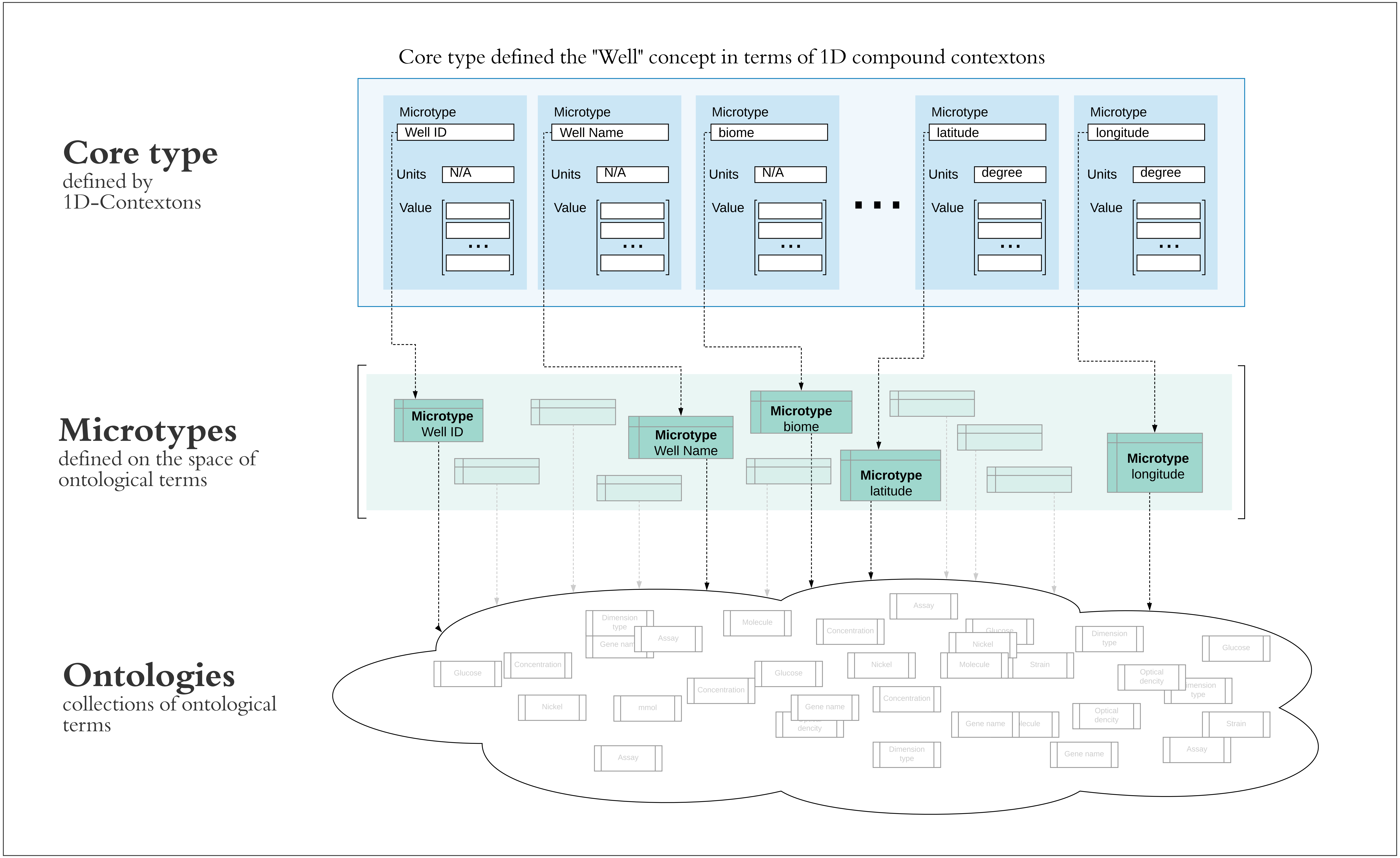

DYNAMIC TYPES DEFINED BY CONTEXTONS AND PRE-DEFINED DATA STRUCTURES

powered by microtypes

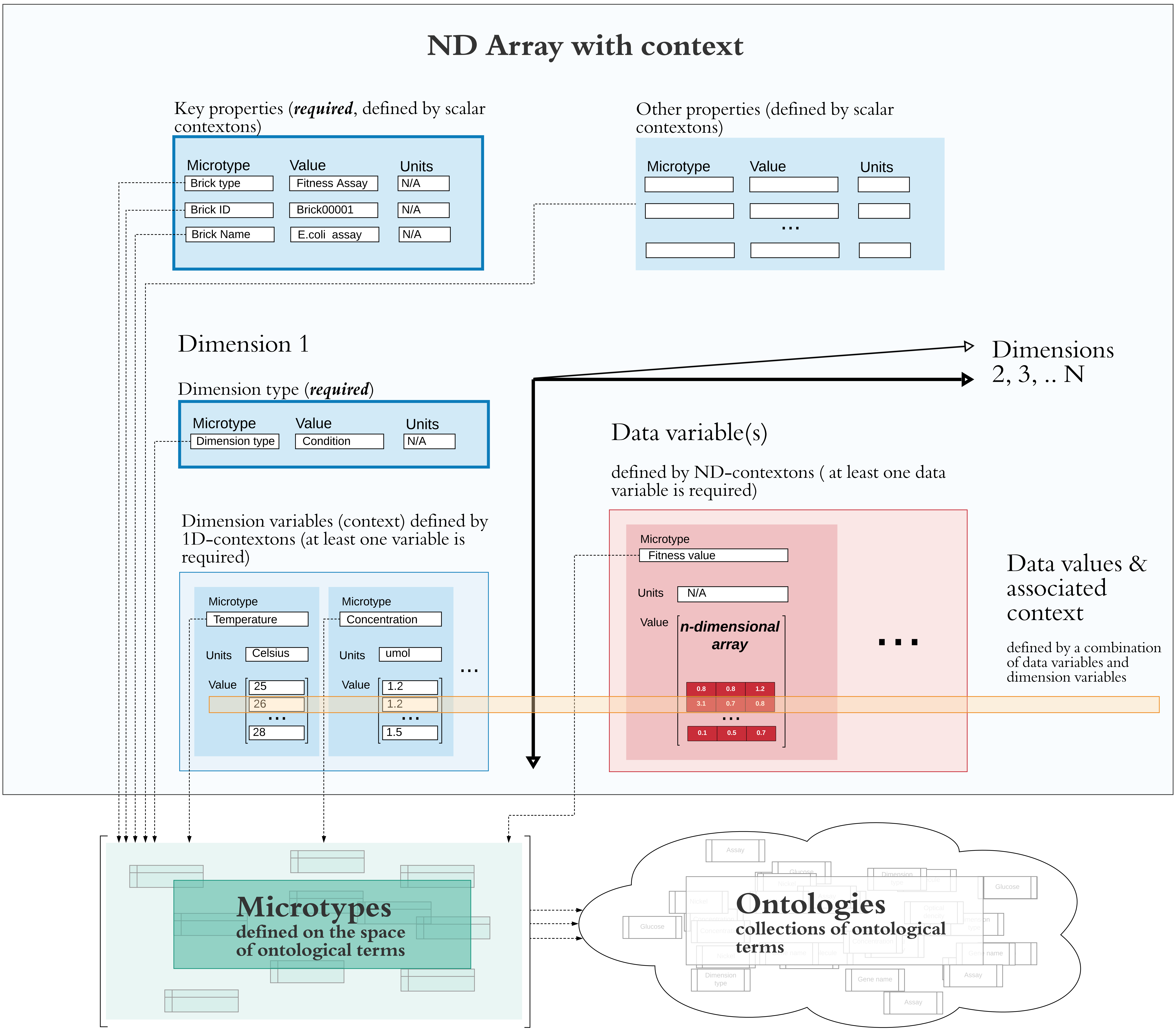

MULTIDIMENSIONAL CONTEXTONS

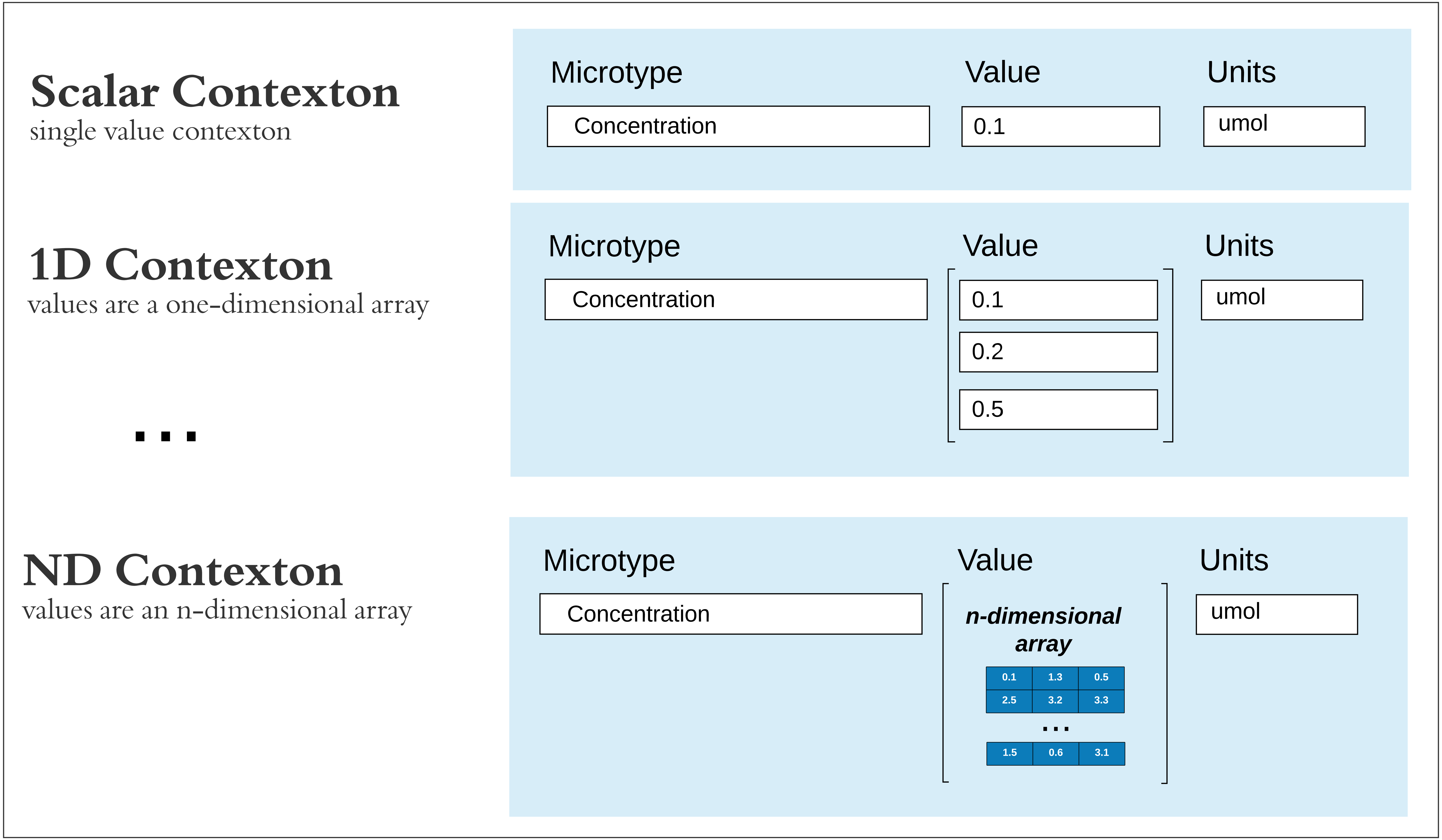

Click here to access/download
Supplementary Material
coral-gigascience-supinfo-v03.docx
