# Supplementary material for: CORAL: A framework for rigorous self-validated data modeling and integrative, reproducible data analysis
Source: Gigascience. 2022 Oct 17;11:giac089. doi: 10.1093/gigascience/giac089 (PMC9575582; doi:10.1093/gigascience/giac089)
Supplement: giac089_Supplemental_File [file giac089_supplemental_file.pdf]

CORAL: A framework for rigorous self-validated data modeling and integrative, reproducible data analysis

Supplementary Information

CORAL UI Features

Provenance Graph

After logging in, all users are shown the provenance graph, which displays an overview of all datasets in the system. Checkboxes on the left side of the graph allow users to filter the display to show only a subset of the data; e.g., data produced by a particular project, lab, or person. Figure S-1 shows the complete provenance graph for a subset of ENIGMA data from a published study [1]; these datasets are included in the repository on GitHub.

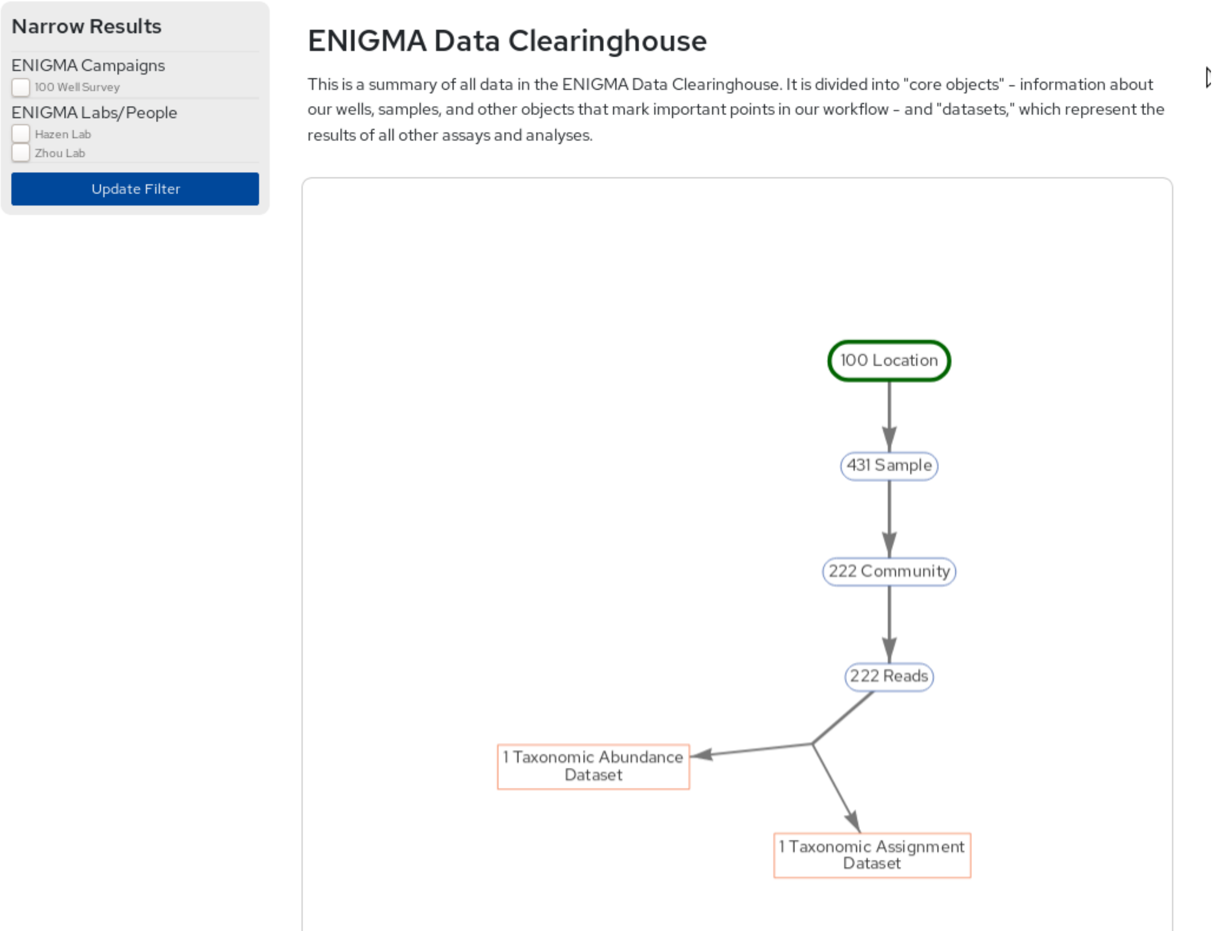

Figure S-1: The provenance graph displays linked data as a directed graph of boxes. Blue ovals indicate static data types, while orange squares represent dynamic data types. Static data types with no external data inputs are shown near the top of the graph and highlighted in green as a starting point for browsing the data. Double-clicking on individual objects brings the

appropriate datasets up in the search interface. Checkboxes on the left side (ENIGMA Campaigns and ENIGMA Labs/People) allow smaller subsets of the data to be shown. If any boxes are checked, only the data produced by a particular lab, project, or person, as well as inputs and outputs, are shown.

Uploader

The CORAL Upload Wizard helps scientists describe their data, and validates all datasets before adding to the system. Steps in this process are shown in Figure S-2, below.

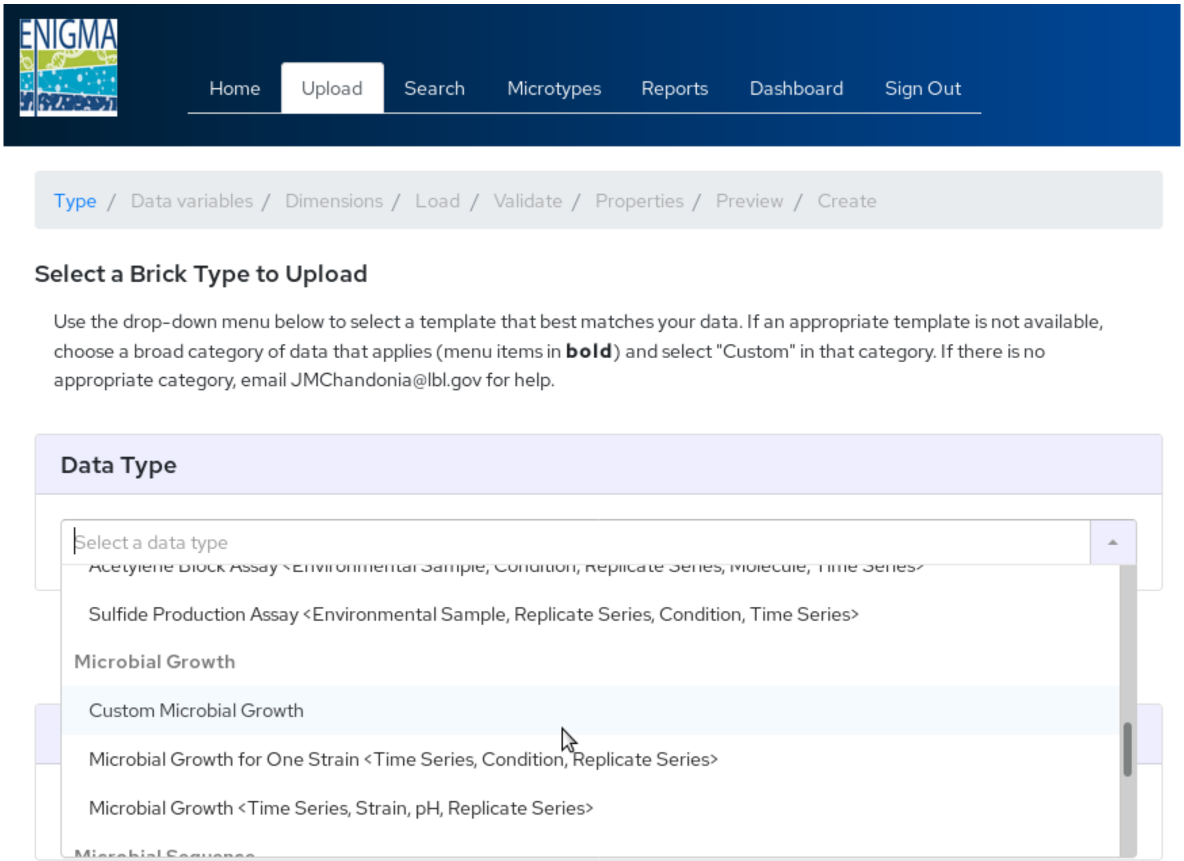

Figure S-2A: Data generators can upload dynamic datasets using pre-defined templates, or build a new data structure on the fly. A list of broad data categories (e.g., “microbial growth”) is defined by the CORAL instance administrators, as are all the possible microtypes and ontological terms that can be used by bench scientists to describe their data.

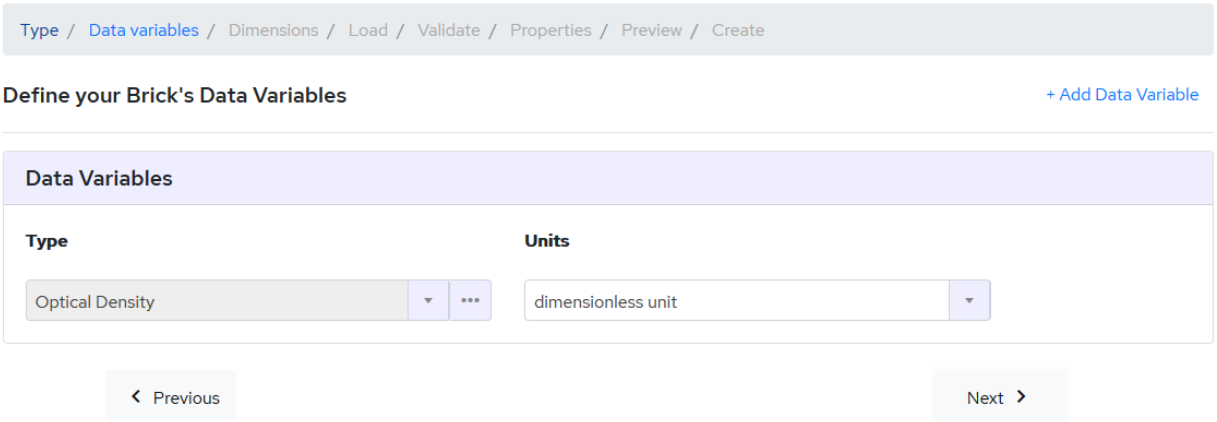

Figure S-2B: Once a data category is chosen, data generators then define the variable(s) stored in their data object. These definitions indicate exactly what quantity was being measured, and the units of measurement. The dropdown for units only displays appropriate units as defined in the microtype. All fields use autocompletion, with a dropdown that displays appropriate microtypes or units that are filtered using the characters typed by the data generator.

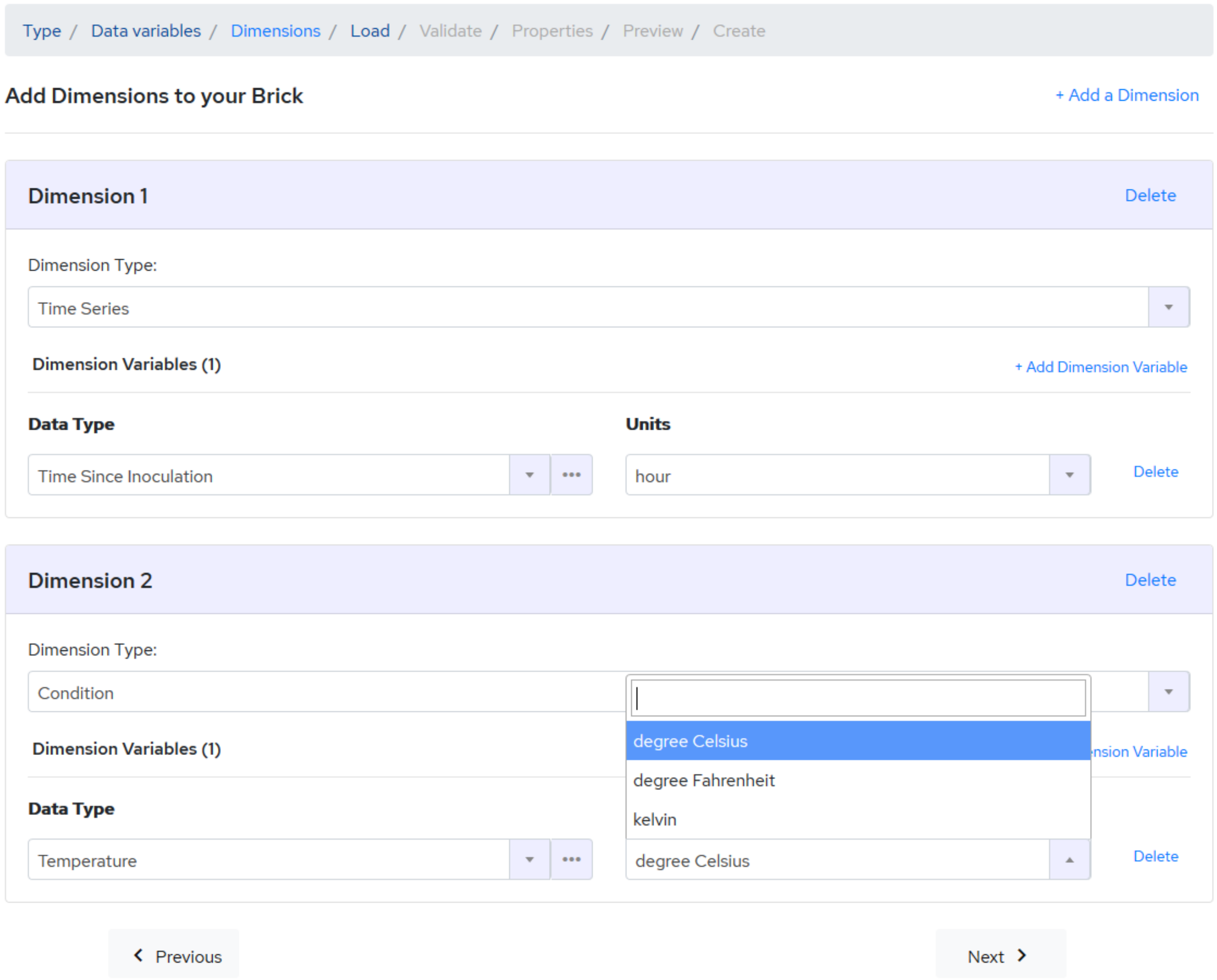

Figure S-2C: Generators next define the overall structure of the data: how many dimensions are in the data, and what varies along each dimension? Generators input these terms using auto-completing text boxes, and valid units for each variable must be selected from a menu. Valid dimension types, and valid units for each microtype, are defined by the CORAL instance administrators.

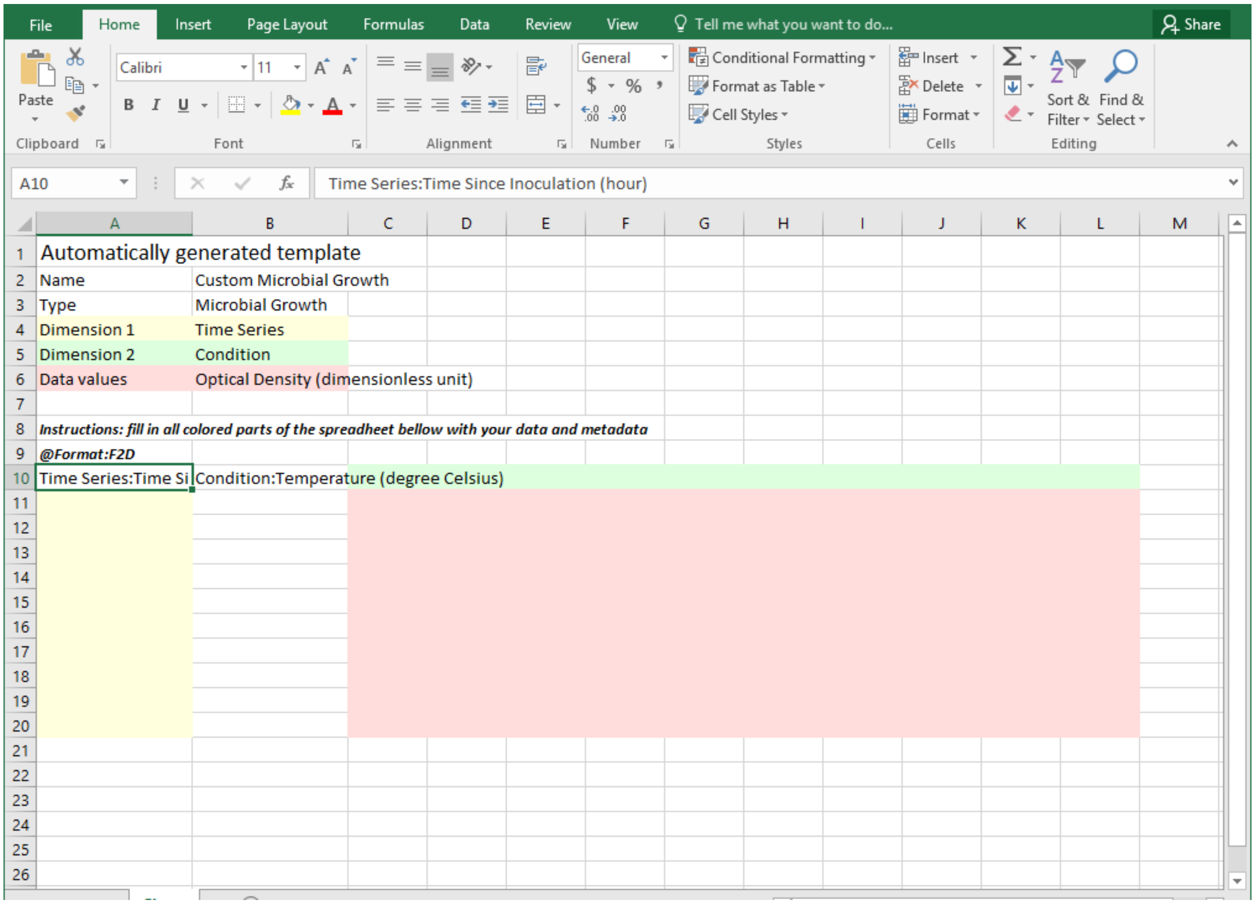

Figure S-2D: CORAL then generates a template spreadsheet into which data generators will paste their data. The template is of the appropriate dimensionality for the data type that was defined in the previous steps. All of the variable names and units are indicated in the template, so the user is clear on where to paste everything.

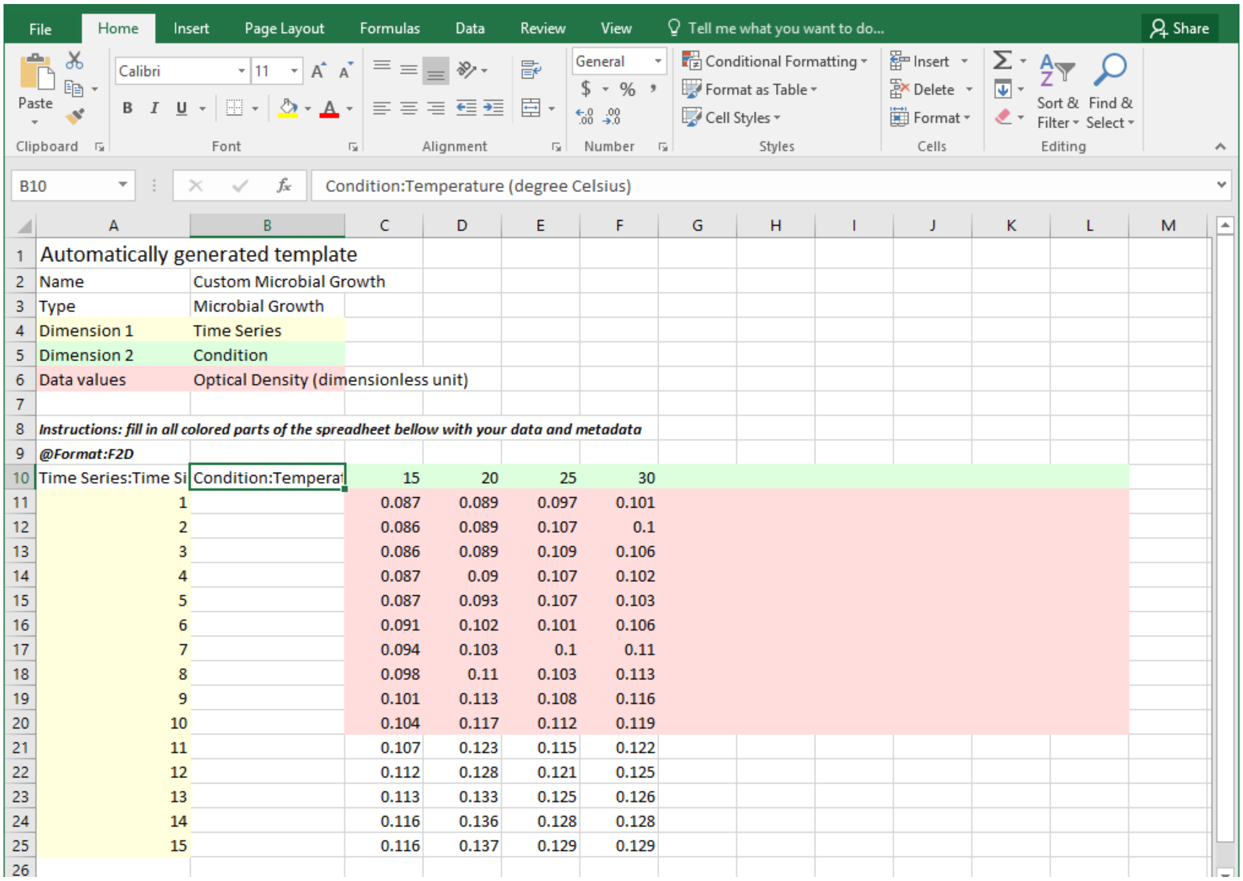

Row	A	B	C	D	E	F
1	Automatically generated template					
2	Name	Custom Microbial Growth				
3	Type	Microbial Growth				
4	Dimension 1	Time Series				
5	Dimension 2	Condition				
6	Data values	Optical Density (dimensionless unit)				
7						
8	***Instructions: fill in all colored parts of the spreadheet bellow with your data and metadata***					
9	***@Format:F2D***					
10	Time Series:Time Si	Condition:Tempera	15	20	25	30
11	1		0.087	0.089	0.097	0.101
12	2		0.086	0.089	0.107	0.1
13	3		0.086	0.089	0.109	0.106
14	4		0.087	0.09	0.107	0.102
15	5		0.087	0.093	0.107	0.103
16	6		0.091	0.102	0.101	0.106
17	7		0.094	0.103	0.1	0.11
18	8		0.098	0.11	0.103	0.113
19	9		0.101	0.113	0.108	0.116
20	10		0.104	0.117	0.112	0.119
21	11		0.107	0.123	0.115	0.122
22	12		0.112	0.128	0.121	0.125
23	13		0.113	0.133	0.125	0.126
24	14		0.116	0.136	0.128	0.128
25	15		0.116	0.137	0.129	0.129
26						

Figure S-2E: The data generator pastes their data (including the values of variables along each dimension) into the template, then uploads the file.

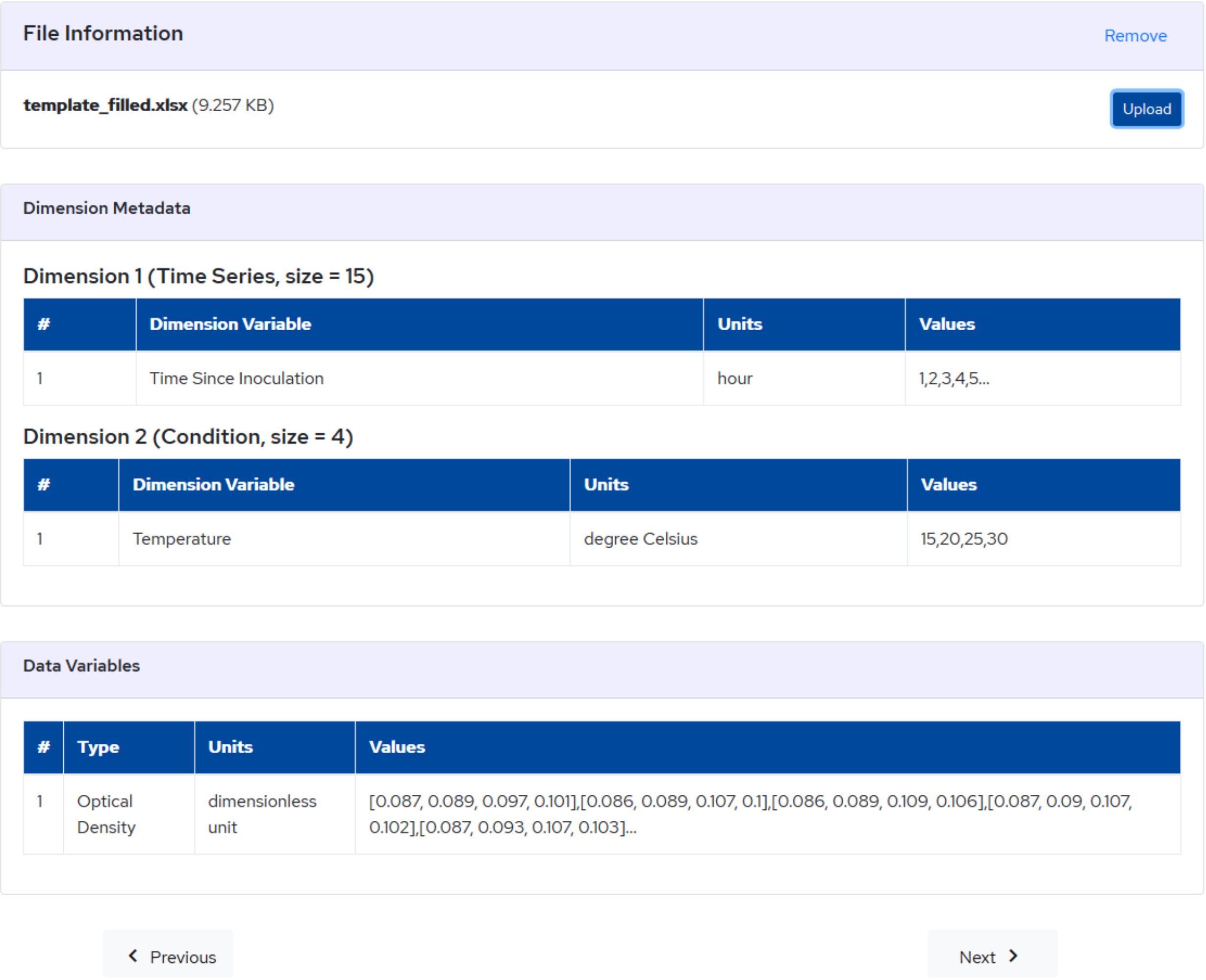

Figure S-2F: CORAL then gives the generator a preview of the data, so they can check whether everything was parsed correctly from the template. Generators may also add additional contextons that describe the context of the entire dataset.

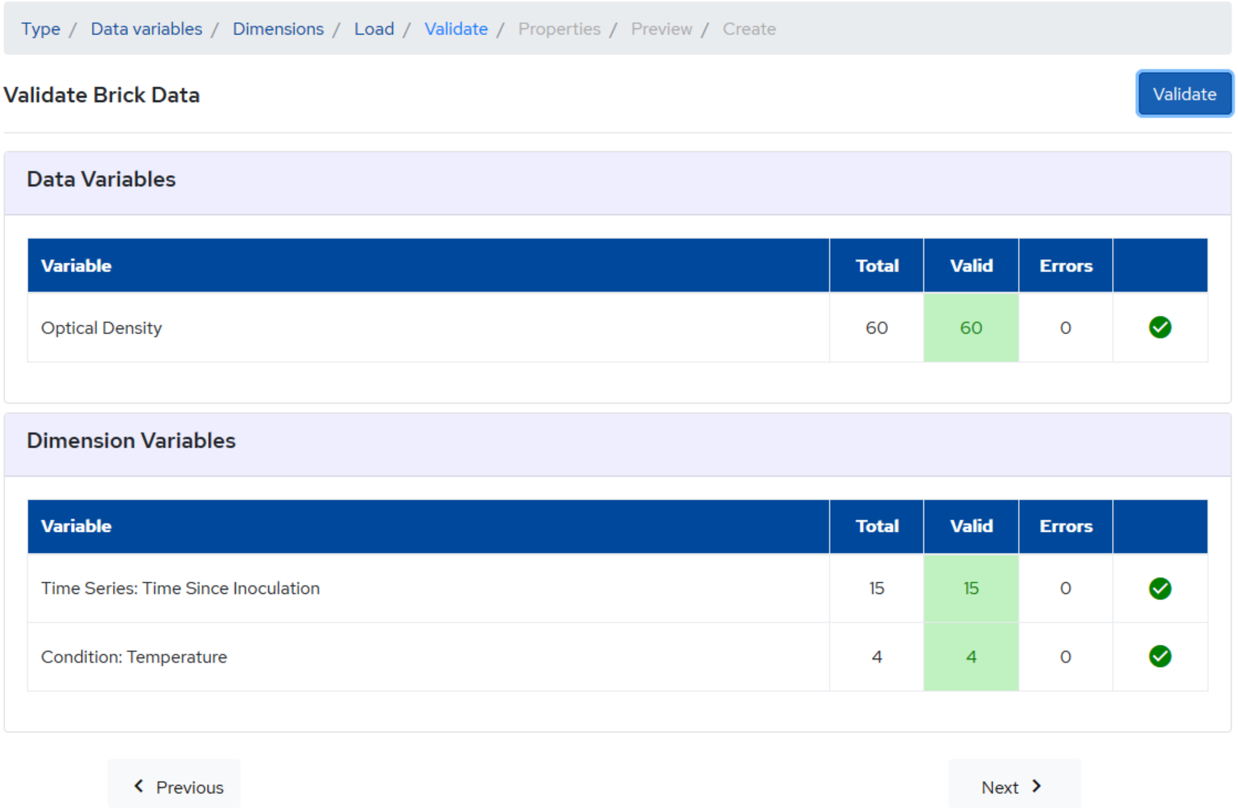

Figure S-2G: Datasets are validated (via validators defined in the microtypes) and linked (via microtypes that refer to other data in the system) before they can be accepted.

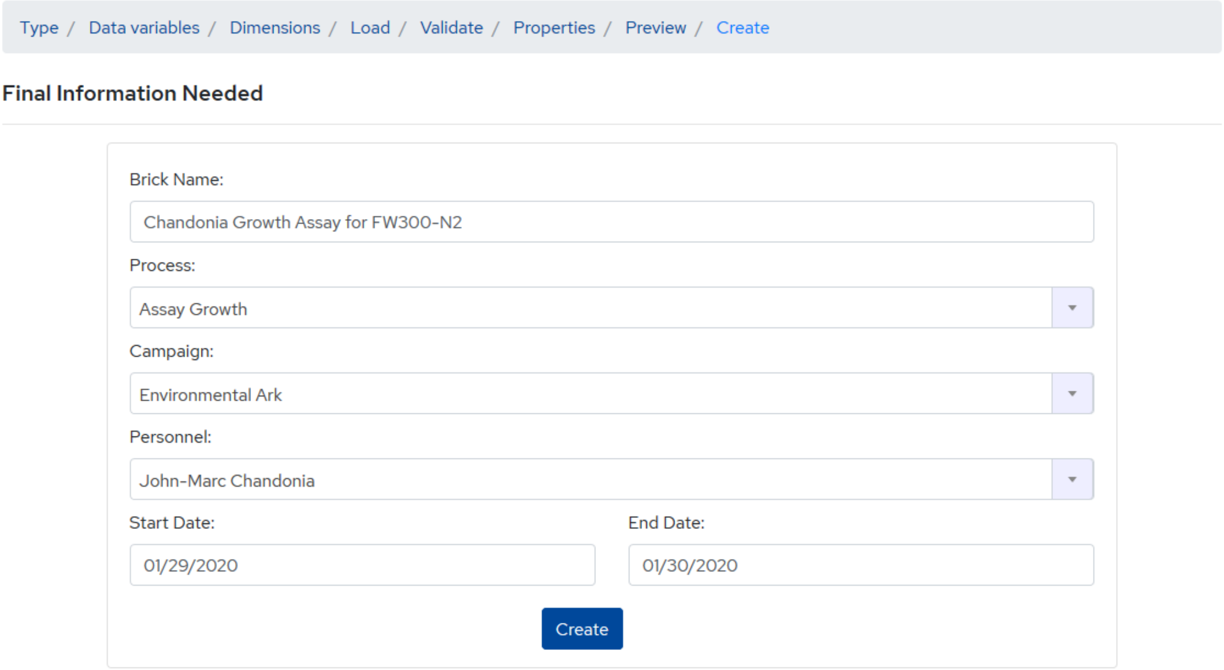

Figure S-2H: As a last step, the data generator provides information about the process by which they created the data, which is used to build links in the provenance graph.

Dynamic Dataset Viewer

After uploading a new dynamic dataset, or finding a dataset through the search interface, users can display an overview of the dataset in the UI. This overview includes all data variables and dimensions, as well as their relevant units, and the process that was used to generate the data. This process information includes the personnel generating the data, their lab, dates, and input objects.

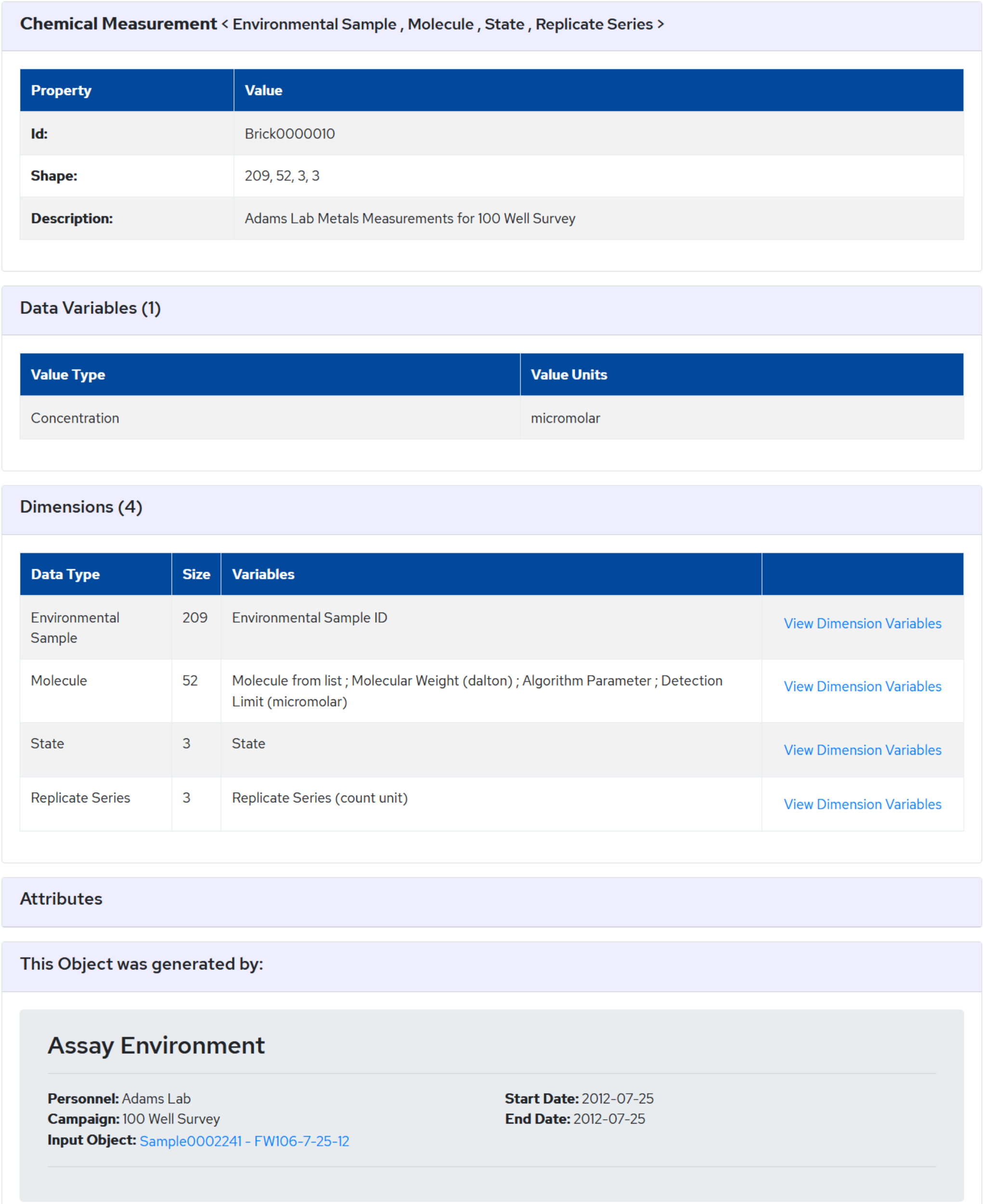

Chemical Measurement < Environmental Sample , Molecule , State , Replicate Series >

Property	Value
**Id:**	Brick0000010
**Shape:**	209, 52, 3, 3
**Description:**	Adams Lab Metals Measurements for 100 Well Survey

Data Variables (1)

Value Type	Value Units
Concentration	micromolar

Dimensions (4)

Data Type	Size	Variables	
Environmental Sample	209	Environmental Sample ID	View Dimension Variables
Molecule	52	Molecule from list ; Molecular Weight (dalton) ; Algorithm Parameter ; Detection Limit (micromolar)	View Dimension Variables
State	3	State	View Dimension Variables
Replicate Series	3	Replicate Series (count unit)	View Dimension Variables

Attributes

This Object was generated by:

Assay Environment

Personnel: Adams Lab
Campaign: 100 Well Survey
Input Object: Sample0002241 - FW106-7-25-12
Start Date: 2012-07-25
End Date: 2012-07-25

Figure S-3: An overview of a 4-Dimensional dynamic dataset containing metals measurements on 209 environmental samples.

Dynamic Dataset Plotting

CORAL includes a wizard to help users plot dynamic datasets. Which plots are available depends on the number of dimensions in each dataset, and the scalar types associated with the microtypes that provide context in each dimension.

If the number of dimensions in a dataset exceeds the number that can be plotted in a given plot type (e.g., a 4-D dataset on a 2-D line graph), the interface requires additional dimensions to be constrained by the user. Each dimension may be plotted as a series, averaged, or a single value from the dimension may be chosen. An example is shown in Figure S-4A.

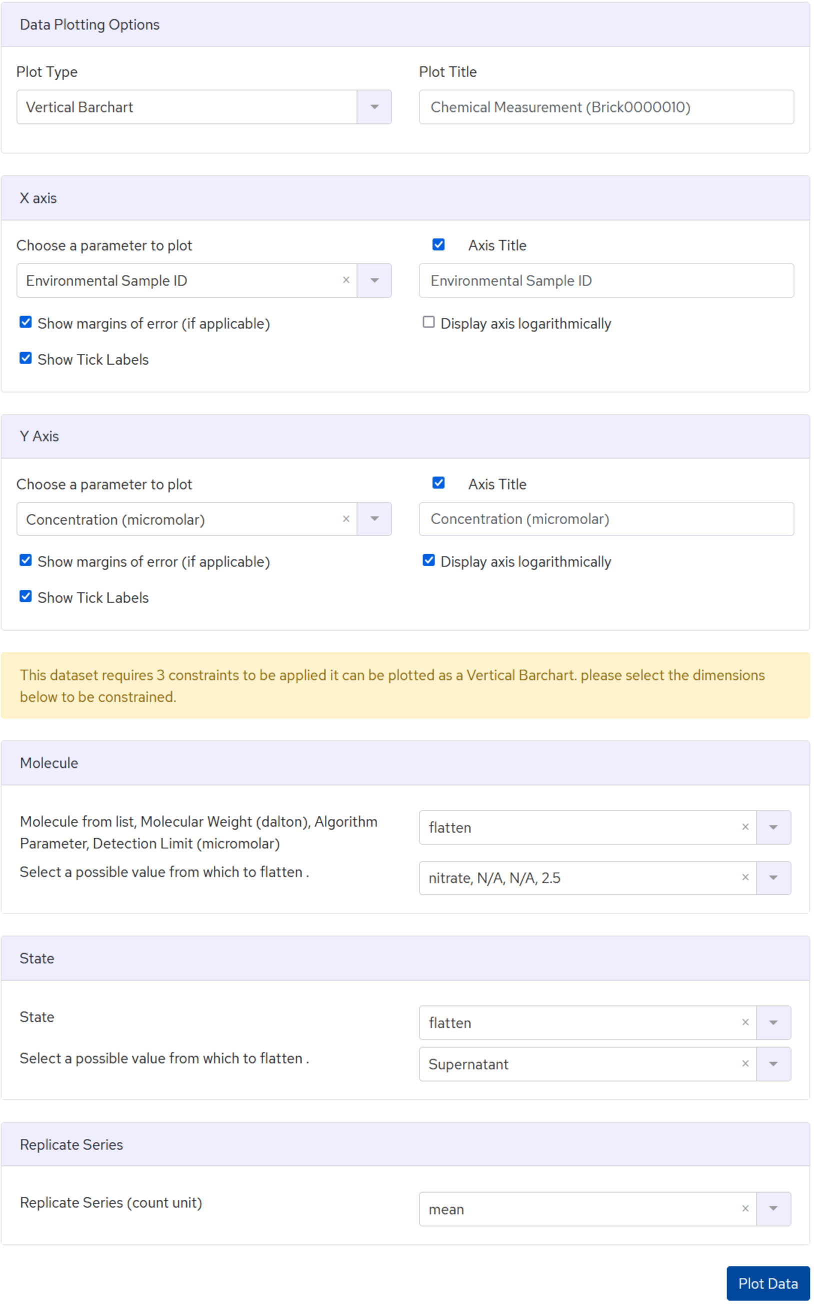

Figure S-4A: A wizard guides the user through plotting a 4-Dimensional chemical measurements dataset as a vertical bar chart.

The resulting plot is shown in Figure S-4B.

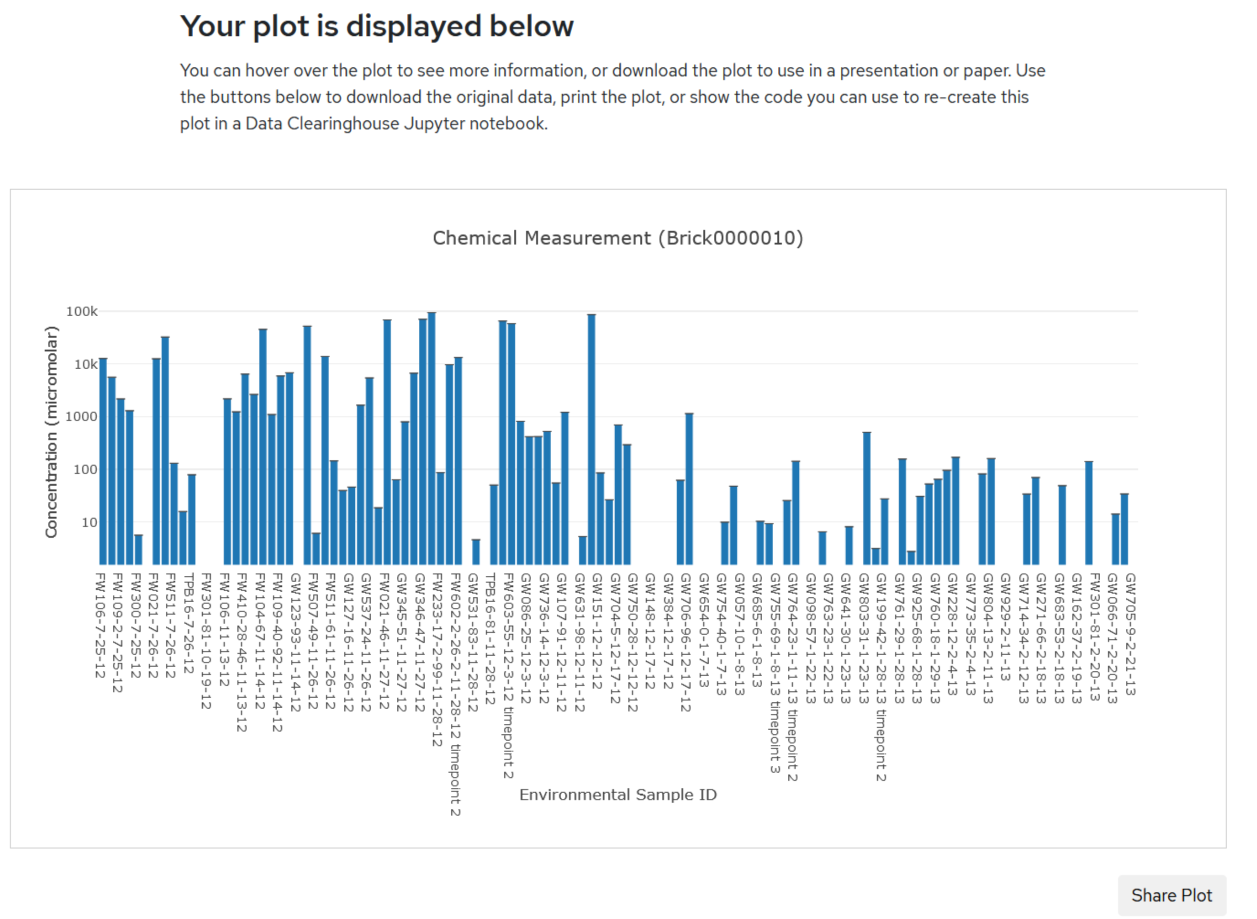

Figure S-4B: An interactive bar chart is shown in response to the user-specified options shown in Figure S-4A. This logarithmic plot shows the concentration of nitrate in a number of environmental samples. The "Share Plot" button allows users to copy the URL, which they can share with other CORAL users to display the same plot again.

Datasets that contain two specific microtypes, *latitude* and *longitude*, may be plotted on a Google Map. Map pins may be colored according to any variable in the data brick. Data that are linked in the provenance graph to a *latitude* and *longitude* may also be projected onto a map. Internally, this process uses dynamic joins (described below) to pull in additional variables from linked datasets; the resulting temporary data brick is then plotted. An example is shown in Figures S-4C and S-4D, below.

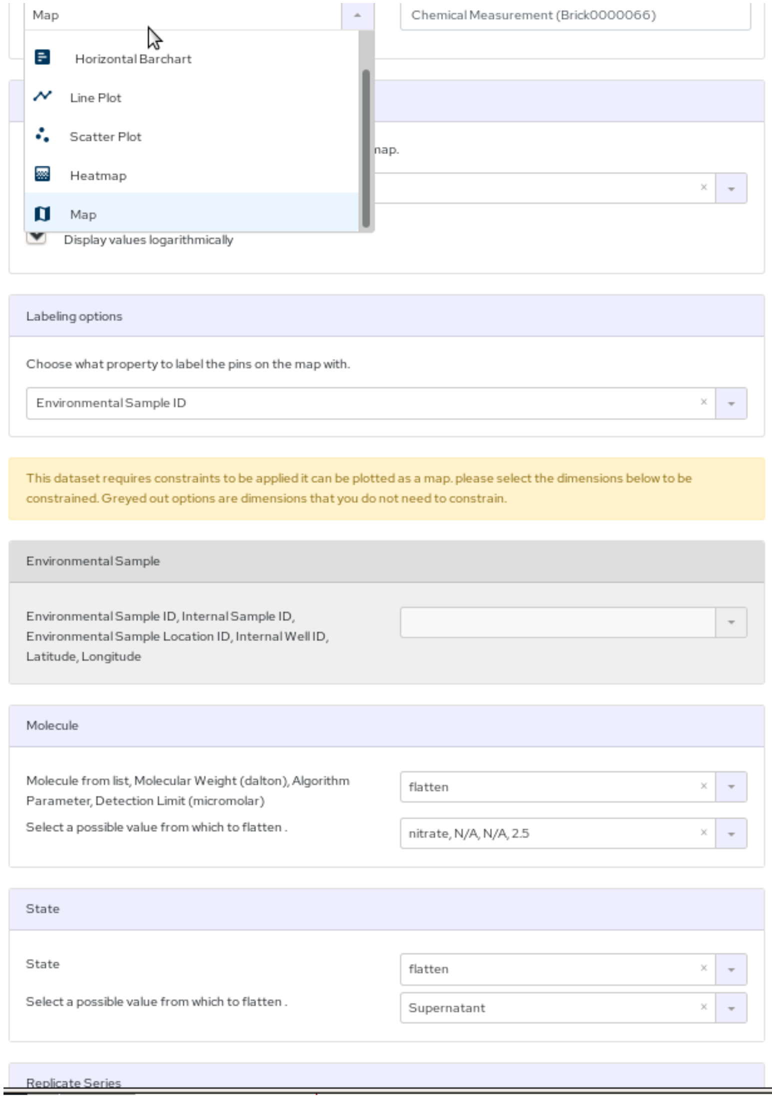

Figure S-4C: An example of the interface for plotting dynamic datasets on a map.

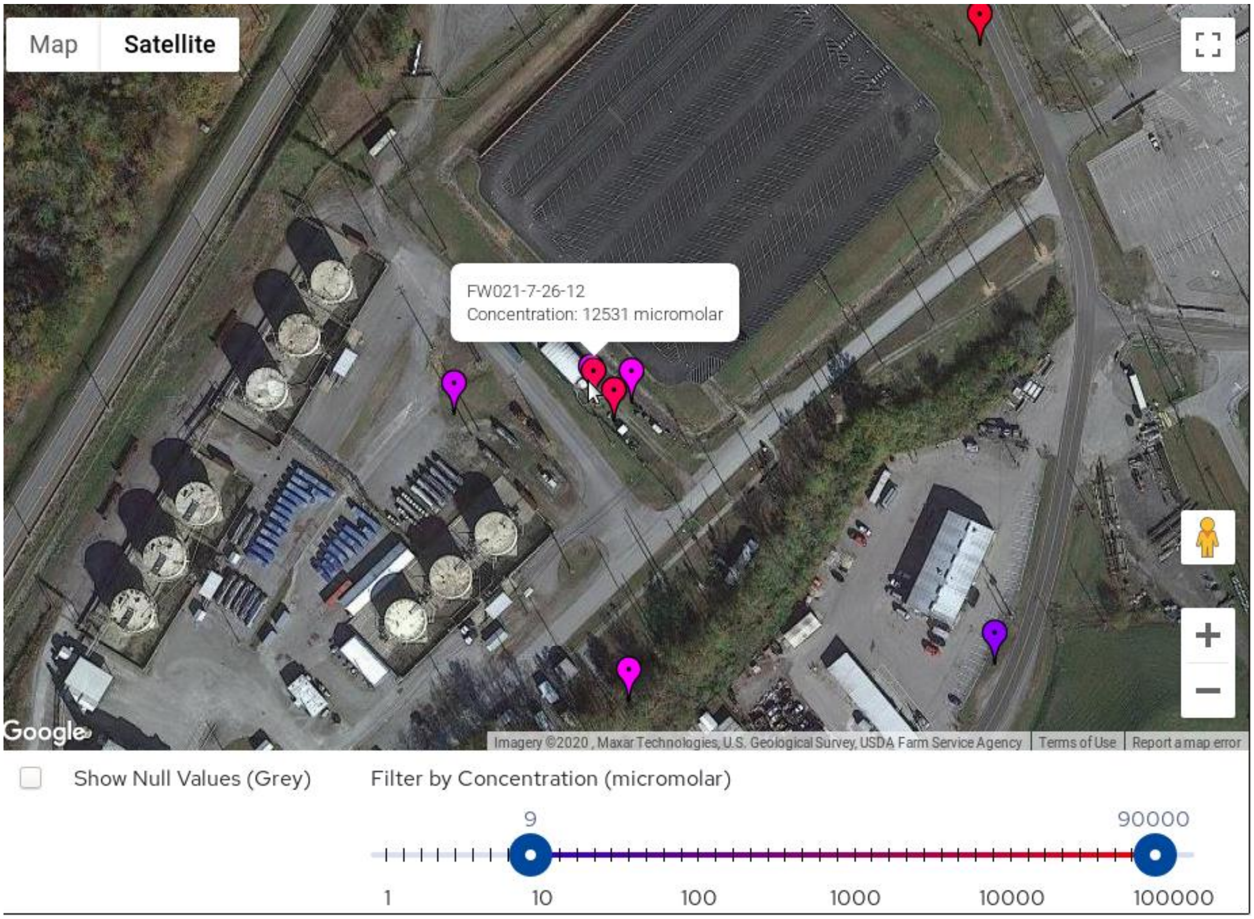

Figure S-4D: An example of the map resulting from Figure S-4C, in which the concentration of nitrate from several samples is plotted on a map, according to the sampling location.

Microtype Tree Browser

To assist data generators and other users with determining which microtypes are appropriate to describe their data, CORAL includes a microtype browser. All microtypes in the CORAL instance are displayed in a hierarchy, which may be expanded by clicking the arrows next to each group. Icons indicate which microtypes can be used to create various types of contextons, e.g., as data variables, dimensions, dimension variables, or properties of an entire dataset. Ontological terms that are not themselves microtypes, but which are used as parents to categorize groups of microtypes, are displayed in light grey. Definitions of microtypes, and their scalar types, are also displayed to the user.

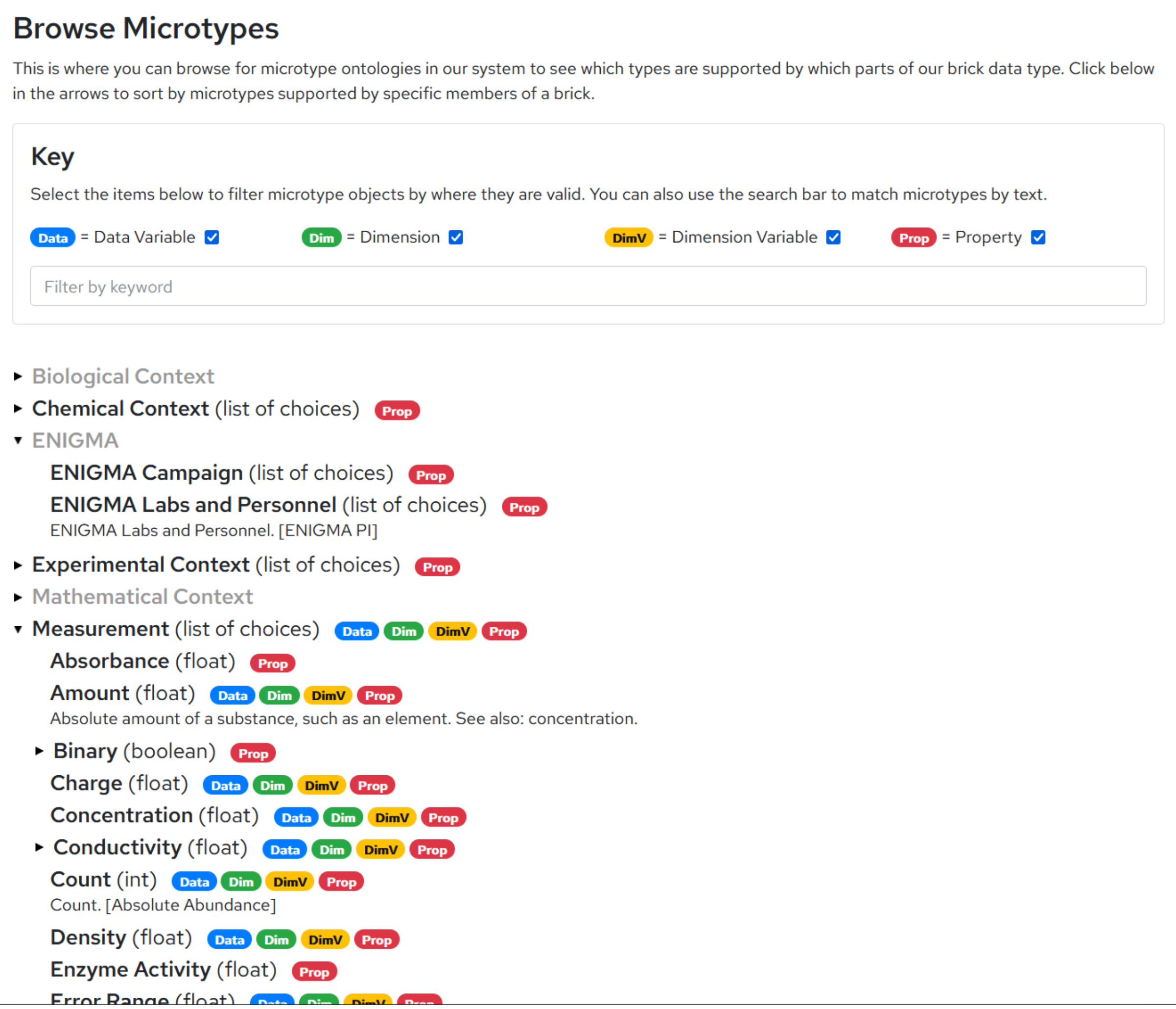

Figure S-5: This figure shows the top level of the microtype tree browser, with the "ENIGMA" category opened to show several sub-hierarchies under it, and the "Measurement" hierarchy opened to show some of the microtypes that represent measurements. The "Filter by keyword" box at the top also allows users to search all microtypes and their definitions; as the user types, the tree is immediately filtered to show only relevant microtypes and their parents.

Compound Contextons

In many instances, context is best described through a combination of atomic microtypes. For example, to express the concept "concentration of nitrate," multiple contextons are combined to build a single *compound contexton*. The primary contexton includes the microtype that indicates the main property being measured (concentration), while any number of additional contextons may be used to modify the first (e.g., indicating that the molecule whose concentration is being measured is nitrate, or that the measurement is taken at 25 degrees Celsius). This example is shown below, in Figure S-6:

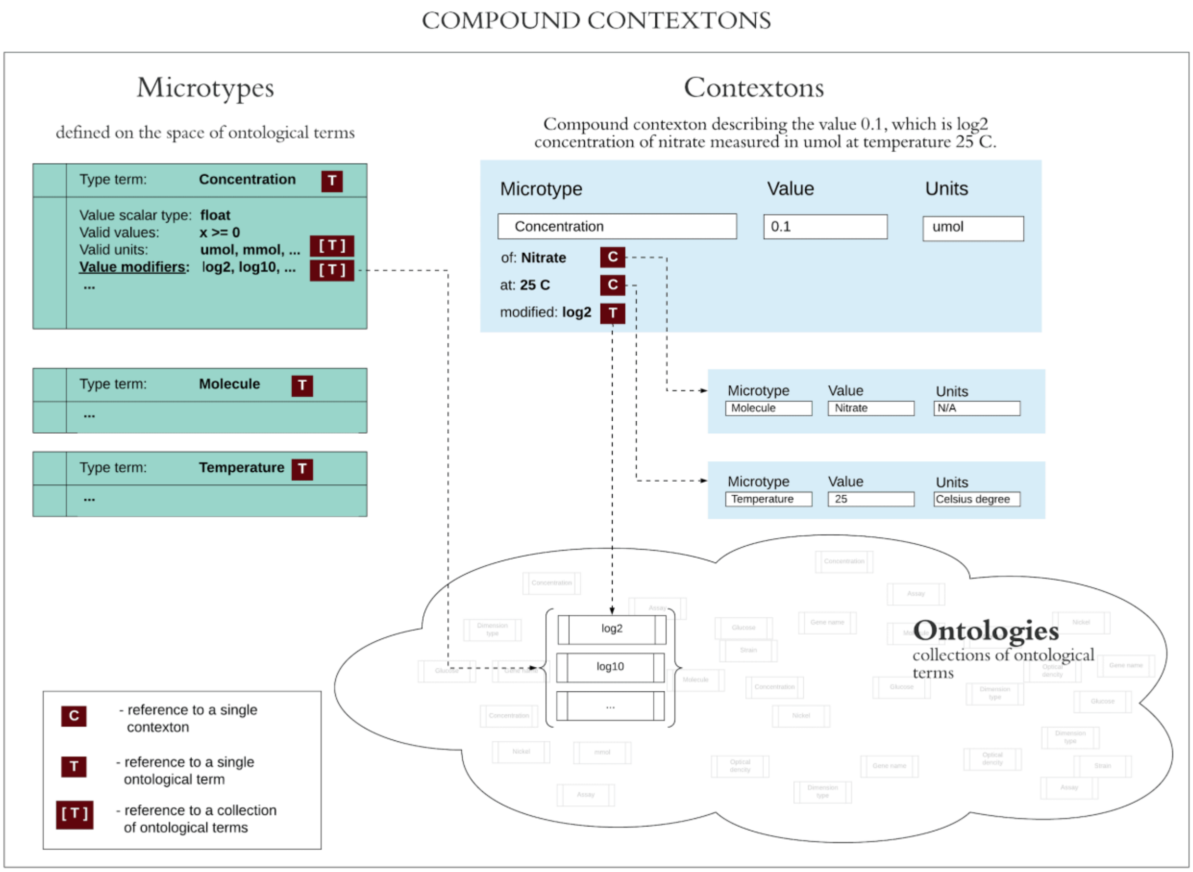

Figure S-6: Example of compound contexton. The Concentration microtype indicates the primary property being measured. Additional modifying contextons indicate that the molecule for which the concentration is being measured is Nitrate, that the measurement is being performed at 25 degrees Celsius, and that the values in the contexton are logarithmically scaled (log base 2).

Dynamic Joins

For example, the dynamic dataset of geochemical data is linked to the Sample core type, and from there to the Location core type. A user wishing to correlate geochemical data with the sampling locations and/or depths could ask CORAL to automatically retrieve and merge all data from the *Sample* core type as additional variables in the dynamic geochemistry dataset. The user could then merge latitude and longitude data into their dataset by using the *Sample* identifiers to map each data point back to the *Well* core type. The resulting larger dataset could be saved as a new dynamic dataset in the CORAL data store, or analyzed further using the wrapped Python libraries.

An example of Dynamic Joins is shown in Figure S-7, below.

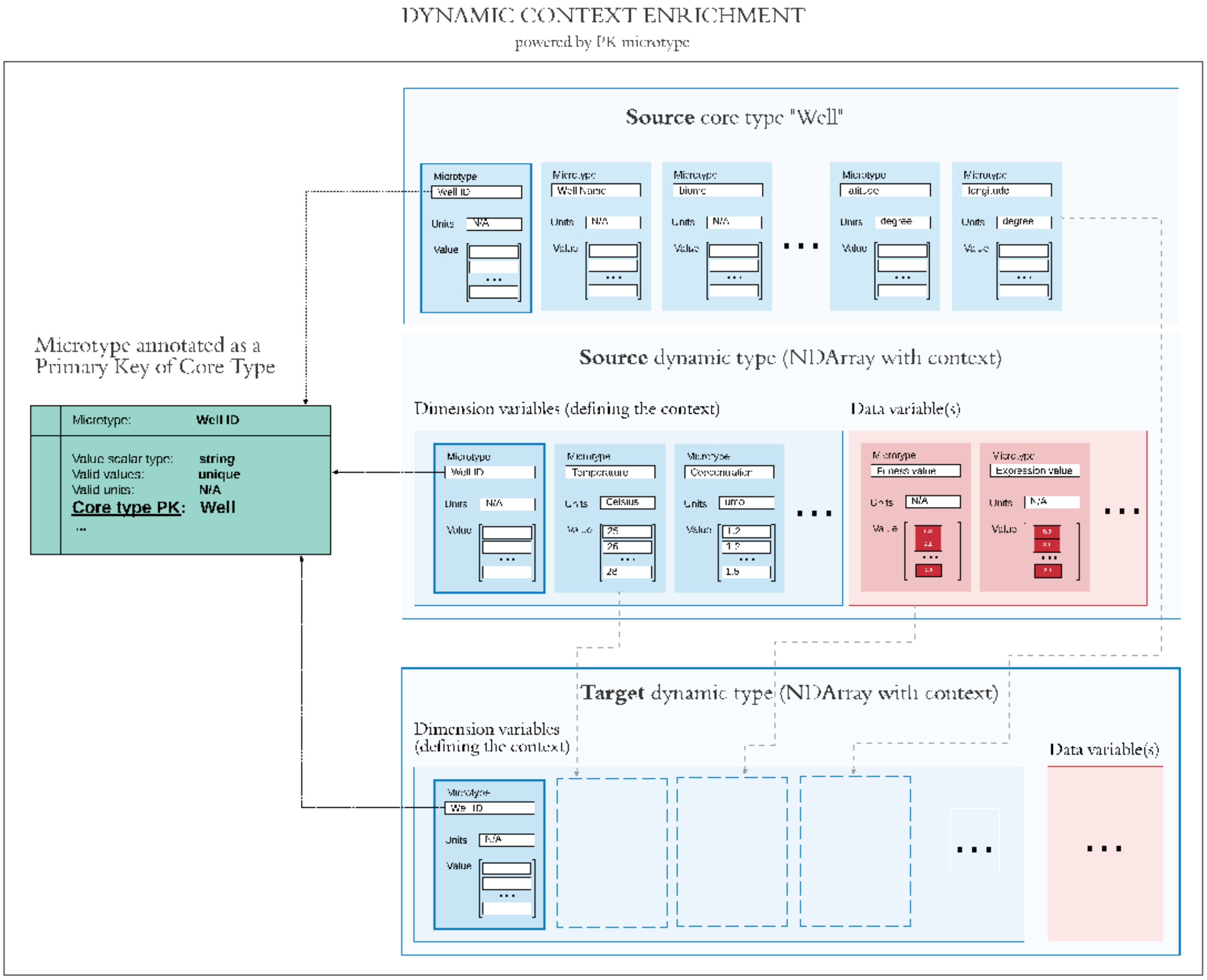

Figure S-7: Example of Dynamic Joins: the user starts with a Source dynamic dataset, which contains data that is linked to the core static type “Well” through the “Well ID” microtype, which serves as a primary key for Well objects. By using Dynamic Joins, the user can bring in fields from the linked (“Source”) Well objects, which become dimension variables in a new dynamic dataset, labeled Target. The Target dynamic object contains fields from both Source objects.

Python API Documentation

The most important aspects of Python API available in the CORAL prototype implementation are described below, and additional documentation is provided in the back end code:

ReportBuilder Service: Creates reports that provide a high level overview of the contents of a particular instance of CORAL.

UserProfile Service: Maintains lists of the microtypes and ontology terms most commonly used by each CORAL user in an instance, in order to increase the convenience of access to these in Jupyter notebooks and front end menus.

DataProvider Service:

find(): implements searches for both static and dynamic data types, allowing simultaneous queries of both the metadata in particular objects as well as their provenance relationships in the ArangoDB graph database.

create_brick(): creates a dynamic data object

load_brick(): returns a dynamic data object stored in CORAL

type_names(): queries the Ontology Service to find all valid Data Categories allowed in this CORAL instance for dynamic data objects.

Arango Service: Provides wrappers for commonly used ArangoDB queries.

Ontology Service: Maintains a database of microtypes and ontological terms, which is backed by a collection in the ArangoDB database. The ontology service also provides convenient methods for searching this collection.

Workspace Service: Includes methods for storing and retrieving static and dynamic objects, as well as processes, using the underlying ArangoDB data store.

Typedef Service: Includes methods for validating the definitions of static core data types, including foreign keys, and validating that new static core data meet the defined standards (e.g., correct scalar types, boundary checking).

Validation Service: Includes methods for validating microtypes, such as checking that the scalar types represented in a contexton are correct, that the units are compatible with the allowed units, etc.

Tools: Provides wrappers for popular Python tools, enabling them to operate on NDArray objects without losing the contextons attached to the underlying numeric data. For example, we have wrapped the sklearn.decomposition.PCA class from the widely used machine learning

library scikit-learn [2], which performs principal component analysis (PCA) on NDArray objects to project them to a lower-dimension space. PCA fitting of a NDArray with a large first dimension results in a new NDArray with the first dimension replaced by a new “PCA Components” dimension of shorter length (based on the number of components requested by the user). All context describing the remaining dimensions is preserved in the PCA output.

Web Services: Serves the REST API (detailed below).

REST API Documentation

Details of the available REST API calls in the prototype CORAL implementation are given below. Full implementation details are provided in the source code on GitHub, in the file back_end/python/coral/web_services.py. API calls are grouped in the following categories:

Remote Data Access

The following API calls are accessible to both logged in users and users who are not logged in, but who possess a valid remote data access token (see main manuscript for details):

/coral/search – Search for data, returning lists of object identifiers for both static and dynamic objects. Queries can be constructed to search within data objects (i.e., the contextons), and/or to search according to the provenance of objects (e.g., objects that link up or down to other objects in the ArangoDB provenance graph).

/coral/brick/<brick_id> - Return a dynamic object (brick) in CSV or JSON format.

/coral/filter_brick/<brick_id> - Returns a subset of data stored in a single dynamic object (brick). Filtering supports simple operations such as slicing on any dimensions of the NDArray object, or averaging all values in a dimension in order to flatten the brick in that dimension. This API primarily supports plotting of dynamic datasets, as described above.

/coral/data_types – Returns a list of all static and dynamic data types in a CORAL instance.

/coral/data_models – Returns the fields (1D contextons) that define all static core types, including scalar types and units if applicable.

/coral/core_type_names – Returns a list of all static data types in a CORAL instance.

/coral/core_type_props/<obj_name> - Returns the fields (1D contextons) that define a particular static core type, including the units of each contexton if applicable.

/coral/core_type_metadata/<obj_id> - Return a particular static core object in TSV or JSON format.

/coral/process/<process_id> - Returns details of processes (edges in the provenance graph).

/coral/types_graph – Returns the complete provenance graph (described above) or a subset of the graph filtered according to campaigns or personnel.

Ontology and Microtype Search

These API calls search microtypes and ontology dictionaries defined in an instance of CORAL. They are primarily used for autocompletion, to map words or partial words input by the users to all matching ontological terms or microtypes. There are separate API calls for each type of *System Microtype* (see main manuscript for definition).

/coral/search_property_value_oterms – Searches all ontological terms and microtypes in an instance of CORAL, according to definitions, aliases, and/or parent terms.

/coral/get_property_units_oterms – Returns all valid units for a particular microtype.

/coral/search_data_variable_microtypes/<value> - Searches *Data Type* microtypes.

/coral/search_dimension_microtypes/<value> - Searches *Dimension Type* microtypes.

/coral/search_dimension_variable_microtypes/<value> - Searches *Values Type* microtypes.

/coral/search_property_microtypes/<value> - Searches all microtypes.

/coral/get_process_oterms – Returns all valid *Process* types defined in a CORAL instance.

ENIGMA-specific API

The prototype implementation of CORAL is customized to the ENIGMA workflow, and thus some aspects of project management, such as the organization of experiments into projects called “campaigns” and the association of each dataset with personnel who created it are encoded in our instance-specific definition of the Process type. These API calls return valid personnel and campaigns, based on instance-specific ontologies:

/coral/get_personnel_oterms – Returns all valid personnel defined in a CORAL instance.

/coral/get_campaign_oterms – Returns all valid campaigns defined in a CORAL instance.

/coral/filters – Returns all valid options for filtering the provenance graph in the UI (e.g., lists of personnel and campaigns to include in the graph).

Upload-specific API

Uploading Dynamic Types

/coral/brick_type_templates – Returns a list of all templates available in an instance of CORAL for uploading dynamic data types.

/coral/generate_brick_template – Generates an Excel format template for users to paste their data into. The template matches the structure of the dimensions and data variables provided by the user.

/coral/upload – After the user has pasted data for a new dynamic dataset into a template spreadsheet, this method uploads it into the system for validation.

/coral/upload_csv – Same as above, but for (expert mode) uploading of CSV-formatted datasets (This is useful for uploading large or automatically generated datasets, as an alternative to pasting data into a spreadsheet template).

/coral/validate_upload – The first step in validating a dynamic dataset that is in the process of being uploaded.

/coral/validate_upload_csv/<data_id> - Same as above, but for (expert mode) validating uploaded CSV-formatted dynamic datasets.

/coral/refs_to_core_objects/ - Scans a dynamic dataset and identifies all contextons that refer to static core objects, then validates that the references are all valid identifiers (i.e., refer to existing core objects in that CORAL instance). This is used during validation of bricks being uploaded.

/coral/dim_var_validation_errors/<data_id>/<dim_index>/<dim_var_index> - Returns a list of errors encountered during validation of a user-uploaded dynamic dataset (for dimension variables).

/coral/data_var_validation_errors/<data_id>/<data_var_index> - Returns a list of errors encountered during validation of a user-uploaded dynamic dataset (for data variables).

/coral/search_property_value_objrefs – Search for static core objects by name and by the microtype that corresponds to the static core type to be searched. This is used for autocompletion of core object names in the uploader UI (described above).

/coral/filter_tmp_brick/<brick_tmp_id> - Analogous to the /coral/filter_brick/<brick_id> call, above, this method allows plotting of bricks that are not completely loaded into the system, but are still being uploaded. This is useful for manual sanity checking of data that is in the process of being uploaded.

/coral/create_brick – The final step in uploading a new dynamic dataset into the system, after the data have been validated.

Uploading Static Core Types

/coral/upload_core_types – The first step in uploading new static core data objects into CORAL. Adding new static data *types* must be done by the instance administrators, but once a type is defined, this allows users with upload permissions to upload new data of a defined core type.

/coral/validate_core_tsv_headers – Validates that the headers in user-uploaded static core data are as expected, based on the typedef configured by instance administrators.

/coral/get_core_type_results/<batch_id> - The final step in uploading new static core data. This method returns a list of warnings and errors encountered during the upload.

/coral/update_core_duplicates – This method allows users to make corrections to erroneous data in core data types. When uploading, if CORAL encounters a duplicate key (i.e., name for a static data object), this method allows the user, though the UI, to assert that they are correcting a dataset that is already in the system.

/coral/get_provenance/<type_name> - Checks whether a static core type requires incoming process links when new data are added to an instance of CORAL. Static core types that are defined as “top level” in the process hierarchy do not need incoming process links, but all other core types do.

Reporting

/coral/reports – Lists all pre-configured reports that are available on the data objects that have been uploaded into an instance of CORAL.

/coral/reports/<id> - Returns one of several pre-configured reports on the data objects that have been uploaded into an instance of CORAL, in table format.

/coral/report_plot_data/<report_id> - Returns one of several pre-configured reports on the data objects that have been uploaded into an instance of CORAL, in a format suitable for plotting.

Authentication

/coral/user_login – API call to begin the user login process, via OAuth2

/coral/google_auth_code_store – Helper function to process OAuth2 logins via the Google API.

/coral/request_registration – Allows users to request a new account, which must be approved by the CORAL instance administrators.

Plotting

/coral/plot_types – Returns a list of supported plot types, allowing the user to choose one in the UI.

/coral/plot_data – Converts the data values in a dynamic dataset into x, y, and z coordinates for plotting using the plotly library [3].

/coral/plotly_data - Formats a dynamic dataset (i.e., “brick”) for plotting using the plotly library [3].

/coral/plotly_core_data – Formats a set of static core data for plotting using the plotly library [3].

/coral/brick_plot_metadata/<brick_id>/<limit> - Returns information about the dimensions, dimension variables, and size of a dynamic dataset (i.e., “brick”), but not the data values themselves. This API call is the first step required to plot a brick through the plotting interface (described above), and returns additional information beyond the /coral/brick_metadata API call.

/coral/brick_map/<brick_id> - Maps data stored in a dynamic data object to labels, colors, and other plot options selected by the user through the plot interface.

/coral/brick_merged_coords/<brick_id>/<limit> - Automatically merges any applicable data variable that is linked to “latitude” and “longitude” contexton information with those values, so that dynamic datasets derived from a static core object that stores location properties may be plotted on a map.

Other API Calls

/coral/brick_metadata/<brick_id> - Returns information about the dimensions, dimension variables, and size of a dynamic dataset (i.e., “brick”), but not the data values themselves.

/coral/brick_dimension/<brick_id>/<dim_index> - Returns information about a specific dimension of a dynamic dataset.

/coral/brick_dim_var_values/<brick_id>/<dim_idx>/<dv_idx>/<keyword> - Enumerates the data values of a dimension variable.

/coral/search_operations – Returns all the supported operators for building search queries, for display in the UI.

/coral/types_stat – Returns a summary of the number and types of all data stored in a particular instance of CORAL.

/coral/set_node_position_cache – Caches the positions of all nodes in the provenance graph, so that the node positions do not need to be recalculated if the same filter settings are re-used.

/coral/delete_node_position_cache – Deletes the cache of positions of all nodes in the provenance graph, requiring them to be recalculated and automatically repositioned.

/coral/dn_process_docs/<obj_id> - Returns all processes in the provenance graph that use a particular static or dynamic data object as an input.

/coral/up_process_docs/<obj_id> - Returns all processes in the provenance graph that use a particular static or dynamic data object as an output.

/coral/microtypes – Returns a data on all microtypes in an instance of CORAL. This is used by the microtype tree browser, described above.

/coral/image – Returns a full-size image or thumbnail, for static core types that are images.

Product Comparison

Many contemporary data science tools include some, but not all of the features of CORAL. Low-level data models (e.g., pandas dataframes, the R language) allow N-dimensional data to be modeled, but don't support the rigorous formal documentation of metadata that CORAL achieves using microtypes. SQL-based data platforms can be made as rigorous as CORAL at documenting this metadata, but those have far higher maintenance costs. Data formats like ISA-Tab allow metadata to be rigorously documented for individual files, but these don't allow sophisticated modeling and annotation of N-dimensional datasets, with formally annotated context in each dimension.

Some more complex tools and databases include some concepts similar to those in CORAL. For example, many biological databases (e.g., Planet Microbe), document metadata using ontological terms or controlled vocabularies, but these databases are specialized for a particular data type (usually sequence data, such as amplicon reads or metagenomic sequences), and do not support nearly the range of data types that can be modeled using CORAL.

Like CORAL, Palantir's open XML data formats clearly annotate the provenance of all data. However, Palantir's datasets are stored as documents (e.g., text documents or spreadsheets) annotated with simple metadata, unlike CORAL's more complex mathematical data types, which facilitate computational analyses of the data.

	Low Setup and Maintenance Costs	Mandates Context	Easy to Evolve new Data Types	Powerful Analysis Tools	Complex Data Structures on which Computations can be Made
CORAL	Y	Y	Y	Y	Y
Python/pandas	Y	N	N	Y	Y
R	Y	N	N	Y	Y
SQL	N	Y	N	N	N
MongoDB	Y	N	Y	N	N
Apache Arrow	N	Y	N	N	N
Apache Parquet	N	Y	N	N	N

Planet Microbe	N	Y	N	N	N
Palantir	N	Y	Y	Y	N

Applications

The most time-consuming and challenging part of deploying CORAL in a new organization is customizing an appropriate set of core types and ontologies that are necessary and sufficient to document a particular business domain. However, once that is done, deploying new data types or modifying existing types is quite easy: only minor additions to ontologies and core types are required.

ENIGMA

Our first application for CORAL is the ENIGMA project, a large consortium of researchers that study how communities of microbes interact with their environment (https://enigma.lbl.gov/). We list the main ontologies, as well as core types, used for this project. Detailed instructions for installing CORAL with a subset of ENIGMA data from a published study [1] are included in the repository on GitHub.

Ontologies

- ChEBI - a public database of molecules and small chemical compounds [4]
- Context/Measurement Ontology - a custom ontology we built for describing the mathematical, physical, chemical, or biochemical context of measurements that are typically taken by environmental microbiologists, as well as a list of terms for such measurements, statistics performed on those measurements, and a list of specialized units relevant to these measurements
- Continents - a custom ontology listing seven continents
- Countries - a custom ontology listing current and historical countries, adapted from http://www.insdc.org/country.html
- Data Type Ontology - a custom hierarchic ontology of data types collected or used by ENIGMA scientists, including all valid types for dynamic types
- Dimension Type Ontology - a custom hierarchic ontology of valid dimension types from NDArrays used to store ENIGMA scientists' data
- ENIGMA-specific Ontology - custom terms that capture some of the ENIGMA management hierarchy, such as PI, scientist, and campaign names
- Environment Ontology - a public ontology used to describe biomes in which samples were collected
- MIxS Ontology - a custom ontology containing environmental terms from MIxS (https://press3.mcs.anl.gov/gensc/mixs/)

- Process Ontology - a custom ontology containing all the valid processes used within ENIGMA. The processes connect the core types (listed below) to each other, and to dynamic types.
- Units Ontology - public ontology for describing units of measurement [5]

Core Types

- Well/Sampling Location - a 2D location that stores the latitude, longitude, and other invariant metadata that describe a geographic location where samples are collected
- Sample - a water or soil sample taken from a Well/Sampling Location, which stores the time, depth or elevation, and other additional details that are specific to a given sample
- Taxon - a reference to the NCBI taxonomy
- OTU - an OTU called from 16S reads
- Condition - an environmental or laboratory condition under which experiments are conducted
- Strain - a named or characterized strain of a microbe
- Community - a group of one or more microbes, grown together
- Reads - DNA or RNA sequences collected by sequencing a sample or community
- Assembly - a set of assembled reads
- Genome - an annotated assembly (i.e., with genes called)
- Gene - a single gene in a genome
- TnSeq Library - a knockout library created for a given strain

Finance

For a company that is tracking financial data on their customers, we would need to design ontologies and core types similar to the following:

Ontologies

- Assets - a custom ontology listing various types of assets, in a hierarchy according to asset class
- Credit Ontology - a custom ontology containing terms relevant to credit reports
- Currencies - a custom ontology with terms for world currencies

Core Types

- Person - records relating to an individual
- Account - records of a person's accounts, e.g., liabilities or debts. Financial records for each account would presumably be linked to this as dynamic types.

Medical

A medical firm may wish to track records related to a person's health, as well as financial records such as billing. Some potential ontologies and core types are:

Ontologies

- Medical - an ontology containing terms related to medical records, tests, anatomy, etc.
- Financial - an ontology containing terms relevant to billing, insurance, etc.

Core Types

- Patient - records related to people who are patients
- Staff - records related to doctors and other medical personnel
- Contact - other people who are not medical staff or patients
- Test - records of medical tests; the results themselves would presumably be stored as dynamic types.

Supplementary References Cited

1. Smith MB, Rocha AM, Smillie CS, Olesen SW, Paradis C, Wu L, et al.. Natural Bacterial Communities Serve as Quantitative Geochemical Biosensors. *mBio*. American Society for Microbiology; 2015; doi: 10.1128/mBio.00326-15.

2. Pedregosa F, Varoquaux G, Gramfort A, Michel V, Thirion B, Grisel O, et al.. Scikit-learn: Machine Learning in Python. *J Mach Learn Res*. 12:2825–302011;

3. Plotly Technologies Inc.. Collaborative data science. Montréal, QC;

4. Hastings J, Owen G, Dekker A, Ennis M, Kale N, Muthukrishnan V, et al.. ChEBI in 2016: Improved services and an expanding collection of metabolites. *Nucleic Acids Res*. 2016; doi: 10.1093/nar/gkv1031.

5. Gkoutos GV, Schofield PN, Hoehndorf R. The Units Ontology: a tool for integrating units of measurement in science. *Database*. 2012; doi: 10.1093/database/bas033.
