# Supplementary material for: CORAL: A framework for rigorous self-validated data modeling and integrative, reproducible data analysis
Source: Gigascience. 2022 Oct 17;11:giac089. doi: 10.1093/gigascience/giac089 (PMC9575582; doi:10.1093/gigascience/giac089)
Supplement: giac089_Response_to_Reviewer_Comments_Original_Submission [file giac089_response_to_reviewer_comments_original_submission.pdf]

Author's Response To Reviewer Comments

Close

Thank you for the opportunity to provide this revised manuscript.

We thank the reviewers for careful review of the manuscript. All changes to our original manuscript were tracked using “track changes.” We hope we have addressed all the concerns, as detailed below:

> Reviewer #1: We thank the authors for their manuscript.

> While the manuscript is well written, I have 2 main comments, one about the manuscript structure and the other about the approach and subsequent claims:

> 1 manuscript structure: I was puzzled by the Methods section, which seems to mainly contain information that would fall under 'Results'. Under "Methods", I was expecting to find information about software engineering practice, user testing and methodology used to assess FAIRness levels and ontology management libraries / components used, as well as information about the language and data standards used for metadata tracking (e.g. W3C prov-o for provenance information, RDF, JSON, JSON-LD in case those had been used.

> Instead, the section contains a long list of components which are specific to CORAL. Can the authors clarify why it is so?

The key novel aspects of CORAL are the concept of a “contexton” and the dynamic data types (i.e., “bricks”) we designed, which use contextons to annotate detailed metadata describing all aspects of numeric datasets. These concepts greatly improve interoperability of datasets produced by different parts of an organization, and thus enable integrative analyses of the datasets. We therefore used the ‘Results’ section, which we renamed ‘Findings’ in this revision, to focus on these aspects of CORAL, which we expect to be most interesting to our readers.

The software itself is a prototype implementation, to demonstrate that this data model is viable and useful in practice. Nevertheless, the prototype software is sufficiently fleshed out to be of potential use to organizations of similar complexity to ours. We therefore used the “Methods” section to document some technical details of the platform, which are in themselves fairly standard software features and do not contribute much to the novelty but would still be of interest to potential users of CORAL.

At the reviewer’s suggestion, we included additional technical details in “Methods” describing some of the ontology management libraries used, provenance tracking, and metadata encoding. Our software engineering and user testing practices are probably not of sufficient general interest to include in the manuscript. We also added documentation of our REST and Python APIs.

Although we have not had the FAIRness levels of CORAL certified by a third party organization, we comply with published principles that are necessary for certification; e.g., https://www.gofairfoundation.org/certification/fair-assessment/

More importantly, the degree to which the CORAL data model enforces ontology-based metadata requirements deep inside the data structure itself (for dynamic data objects) is novel, and therefore exceeds the requirements of the current FAIRness certification processes that we are aware of.

> 2. the approach and claims:

> The authors describe a compositional approach to matrix definitions, relying on "static core types' and 'dynamic type' to define data bricks , why may be reused and shared.

> Key elements (Data Category, Dimension Type and Unit) must be tagged with an ontology term.

Does Coral come with preloaded/default resources/namespaces for those?Data Category, Dimension Type and Unit must be tagged with an ontology term.

CORAL includes a set of default microtypes and sample data used by the ENIGMA project (see the examples/ directory of the github repo), which could be easily adapted for use by other environmental microbiology projects. Our data model is quite general-purpose, as detailed in the Supplementary Information, it might be used for applications as diverse as health care and financial services. It would require extensive domain expertise to configure a set of microtypes and ontologies for such applications, but would nevertheless require less investment than traditional data management methods that yield similar Interoperability and Reusability to CORAL.

> A number of projects have explored these approaches (e.g. CEDAR for metadata description) and ran into the combinatorial explosion of entities. It would seem appropriate for the authors to include a section detailing how to CORAL system help with the status quo?

We added a citation to CEDAR, which is a great solution for managing metadata at the dataset level. However, the problem CEDAR solves is only one aspect of the CORAL data model: dataset-level metadata are analogous to the contextons we use to provide overall context for each dynamic dataset. In addition to that important metadata, we also require context to be provided for each dimension, and for the data values themselves. The latter two requirements ensure the resulting data bricks are computable without losing formal context.

Regarding the “combinatorial explosion” problem, CORAL addresses this through microtypes: using a limited set of microtypes, users can describe hundreds of more complex data objects. Compound contextons also address this problem, avoiding a combinatorial explosion in the number of microtypes themselves as has been seen in other projects such as ENVO (e.g., ENVO has many “concentration of chemical X” terms, which we address by combining two microtypes as discussed in the manuscript).

> In keeping with this topic, a number of questions can be raised:

> If 'data category' must be set from terms coming from an ontology the selection of which is down to system administrator/ data stewards, how does this help interoperability in the absence of community guidelines or queryable registry?

CORAL aims to solve the problem of interoperability within a moderately sized organization, or a set of organizations that agree to a common data model defined on a set of community-defined microtypes and ontologies. For example, all environmental microbiology projects funded by the Department of Energy’s Office of Biological Energy Research might collaboratively agree to a common set of microtypes. While CORAL would thus provide interoperability between these organizations, and therefore incentivize adoption of such common guidelines, features like a queryable registry that would help in the formulation of such guidelines are currently outside the scope of our prototype implementation. We also note that there are public ontology registries users could 'subscribe' to in order to ensure better global interoperability.

> - Does Coral come with preloaded/default resources/namespaces for those?

Yes, the ENIGMA example described above. This includes microtypes, ontologies, and typedefs for many of our core static objects.

> - Will Coral provide 'community annotation profiles'?

Those are outside the scope of our current prototype. We encourage communities to adopt a set of common microtypes in order to enable this.

> - If so, what provisions are made to account for versioning of ontological resources?

The current CORAL prototype could readily be extended to track the version of each external ontological resource used (including microtypes and other internal CORAL configuration). Tools already exist to map terms between versions of resources, and these could be extended to handle microtypes as well.

> a number of approaches exist json schema + ajv validator, rdf metadata profiles (shacl + shex

expression for validation) . These are not cited or mentioned in the manuscript. Have these been explored in the CORAL work and how they could interface with the approach of microtype definitions?

We did not use these approaches to define or validate microtypes. The benefits of RDF would be clear for public-facing CORAL instances, so this is a direction we might explore with future development. CORAL is currently optimized as a platform for storing and analyzing data within an organization, rather than on providing datasets to the public. It is also unclear how RDF could be used to described finer structures of numeric datasets (e.g., dimensions and dimension variables).

> Also, a project such as Biolink.LinkML has made interesting developments, even if at possibly a different level of metadata profile definition, its anchoring in the bio/omics data context could warrant mentionning.

Compatibility with LinkML is worth exploring in the future, as it is similar to our typedefs for static core data types.

> -page 9: "In practice, only a small fraction (~10%) of data types need to be statically defined. " How did the authors come to this estimate? what was the method used?

We performed a survey of data types used by ENIGMA researchers in ~50 studies generated by more than 10 different laboratories over a period of several years. Of 384 data types we found, ~10% were sufficiently complex that there are already specialized formats to represent them (e.g., genome sequences and annotations, microarray data). These would be represented in CORAL as static objects. Of the rest of the data types, most are essentially tables of numeric measurements, and therefore are readily represented as NDArray dynamic objects. We also found some data types that could be equally well represented in either static or dynamic objects, and the benefits of one or the other model would be determined by data managers for a project. We expect that this result is generalizable across most fields of research (and non-research endeavors) that deal primarily with numeric data. Several such examples (e.g., financial data, medicine) are explored in the Supplementary Information.

> -page 10: the authors introduce the notion of "data brick", on top of "microtypes" and "contextons", but this appears nowhere in any of the figures, except in Figure 5 where microtype fields are denoted "brick name", "brick ID" when describing an N-dimensional array.

"Data brick" is synonymous with "dynamic dataset" and these are instances of "dynamic data types." The only figure this is applicable to is Figure 5.

> providing a figure more clearly detailing the relationship between these concepts would help the targeted audience.

We would welcome suggestions as to how to improve Figure 5, which describes how dynamic datasets are built on top of microtypes and contextons.

> - Are brick ID resolvable (over https)?

Yes, using the Remote Data Access API. We have added more details of this in the main manuscript (Methods > REST API), and documented the full REST API in the Supplementary Information.

> Terms of use and license of microtypes, contextions and brick: providing a example of how to do this would be a nice addition

The example microtypes we provide are part of our github repo, which is available under the AGPL. The current prototype implementation stores sets of microtypes in OBO files, and it is common practice to store licenses as remarks in those files. We agree with the OBO Foundry in recommending the use of CC-BY or public domain (CC0) licenses for ontologies and microtypes. Data (e.g., bricks and contextons) are also often released under similar licenses. The brick format allows microtypes to be defined by CORAL instance administrators, so defining a "license" microtype would be a simple way to convey license information for bricks that are placed on a public-facing website. We would consider making such a microtype a mandatory system property if we optimize CORAL for use in public-facing data repositories.

> Figures:

> 5 figures are devoted to the description of the paradigm. I would recommend condensing those into a synthetic one and use the freed real estate to show case other functionalities (wizard, search interface, visualization) which characterise CORAL.

All of the requested figures showing functionality are currently included in the Supplementary Information. Figures 2-5 in the main manuscript are necessary to describe the concept of a data brick; we feel it would be confusing to present Figure 5 without first introducing the prerequisite concepts in Figures 2-4.

> Reviewer #2: The paper introduces a platform called 'Contextual Ontology-based Repository Analysis Library' (CORAL), which provides support of FAIR data principles, with a particular focus on making heterogeneous data interoperable and reusable using an ontology-based approach.

> The framework provides users the facility to add new custom datatypes to extensively document the context of all data based on concepts like microtype, contexton, and ontologies. In addition to this main feature, it also provides support for documenting and uploading datasets, search datasets, plotting wizard for visualization, API access, management dashboard, and Jupytet notebook access through JupyterHub.

> The authors have looked into a relevant area by developing a platform that supports FAIR principles to users with the additional benefit of having little maintenance effort. The authors have clearly defined the challenges in supporting FAIR principles in platforms, with discussing on the greater difficulty in the Interoperability and Reusability principles. The text is well written.

> The authors provide the code for the system with AGPL license. They also provide installation instructions for CORAL, with separate instructions for frontend and backend part.

> Please find below the comments and questions to strengthen this paper:

> * The related work is limited. The authors point out only two related software. However, there are tools that provide FAIR support, particularly providing support for adding new custom data types for adding metadata using ontologies. One such example is The CEDAR Workbench: An Ontology-Assisted Environment for Authoring Metadata that Describe Scientific Experiments, 2017. The authors should provide more examples and discuss on comparing CORAL with existing tools used by the community.

We added a citation to CEDAR, as discussed in our response to Reviewer 1. We also compare CORAL with a number of other similar platforms and libraries in the “Product Comparison” section of the Supplementary Information. As CORAL’s focus is on data management and analysis within a moderately sized organization, the tools for managing metadata constraints on public-facing datasets (e.g., RDF/ShEx/SCHACL) are not currently comparable – although we would integrate such approaches if we adapt CORAL to this use case.

> * On page 6, the authors mention the survey conducted in the ENIGMA project. In addition to providing the project's website, the authors should also directly point the readers to the resources related to the survey, e.g., survey results.

In the Supplementary Information, we describe data from a published ENIGMA study (Smith et al, mBio. 2015; doi: 10.1128/mBio.00326-15) for which the data are included in our github repo. Many additional ENIGMA publications are available on the project’s website, but because we did not mention those data in this CORAL manuscript, we did not cite those publications.

> * The authors mention about ontolgies. However, a definition of the term is missing in the article. The definition of an ontology is provided in Knowledge engineering: Principles and methods. Studer, et al. (1998)

Thank you for the reference – we have included it.

> * It is not clear how the software was designed and developed? How are the requirements needed to develop this platform collected? The authors need to provide more information on the experiment design.

The CORAL data model was driven by our need to model hundreds of data types in a FAIR way, to support integrative analyses across data generated by multiple labs. This is discussed in the last paragraph on p. 8.

The user-facing features of CORAL, discussed in the “Functionality” section on p. 13, were driven by a need for decentralization in data management: in order to prevent bottlenecks, we needed data providers to describe and upload their data in a FAIR format. Thus, the upload and plotting wizards. Our reasoning for why we need this (preserving information as data flows between groups) is summarized in the “Discussion” section.

> * On page 8, the authors mention that the designers of microtypes should endeavor to use community standard ontologies wherever possible: How do the designers ensure that? Are there guidelines for the designers? How are these ontologies selected? Which are the ontologies currently selected in CORAL for this purpose? The authors should provide more explanation on this point as the platform itself is based on an ontology-based approach.

We cannot ensure that users choose community standard ontologies, but not doing so – reinventing similar ontologies from scratch – would be considerably more work.

The ontologies ENIGMA uses are discussed on p. 25 of the Supplementary Information. Those (ENBO, ChEBI, PATO) are standards in environmental microbiology and are used by other projects such as NCBI and MG-RAST.

Researchers in other fields (or people using CORAL outside of scientific research) would need the domain knowledge to choose appropriate ontologies.

> * On page 12: The authors talk about determining the provenance of the data? It would be good first to give a definition of provenance and also to mention, which provenance information is collected in this system?

We added a succinct definition of provenance (“the inputs and methodology used to produce data”) on p. 12. Provenance is stored in the “process” core type, which represents edges in the provenance graph (see caption to Fig. 2). The fields in “process” are defined by the instance administrators, and can be as sparse as just collecting the inputs and outputs of every dataset, or they can be required to collect more information, such as dates, methods, and personnel involved. The example typedef for “process” included in the CORAL github repo includes all of the latter items.

> * On page 14, the authors mention that the microtype names, as well as other ontological terms, are arranged in hierarchies? Generally, in ontologies, each concept has superclasses and subclasses. How is this hierarchy of the ontologies used and visualized to users in CORAL? Do the authors also make use of the reasoning power provided by ontologies in their system? If yes, the authors need to provide that information to these questions.

We added a screenshot and description of the Microtype Tree Browser to the Supplementary Information, in order to show the hierarchy, user interface, and some examples of class relationships.

We make use of the reasoning power of ontologies in several ways. First, we allow searches by parent or child term within the hierarchy. For example, a user looking for data on metal-related experiments could search for the ChEBI term for “metal atom” and find all the datasets in an instance of CORAL that referred to different metals that are subclasses of that term in ChEBI (“aluminum atom”, “lead atom”, etc). We provide one such example on p. 21. Second, we allow restrictions on values and units of microtypes to be specified as superclasses, meaning that any subclass underneath is a valid option. We added such an example on p. 16.

> * On page 15, the authors provide several benefits of the CORAL approach. The information on how the data is stored is currently is missing. Since there is extensive use of ontological terms, is the data stored as Resource Description Framework (RDF)? If yes, please provide more information on this. If

not, please specify how the data and the provenance information are stored and queried by the user?

We added an “Implementation” section on p. 19. to discuss these details. To summarize, most data, including ontological terms, are stored in collections in an ArangoDB database. Dynamic datasets (bricks) are stored in NetCDF-4 format on the filesystem and indexed in ArangoDB.

> * On page 17, the authors mention about the validator to limit the values for a microtype. For example, 'Chemical' microtype. A 'Chemical' can appear in multiple ontologies. In that case, who selects which ontologies to be shown for a microtype. If multiple ontologies are shown to the user, how can the user select which term to select from multiple ontologies?

The instance administrators define which ontologies are included in a particular instance of CORAL. For example, the admins could define ‘Chemical’ to point to ChEBI, or any other ontology of chemical terms. They would point to ChEBI by specifying that allowed values of ‘Chemical’ are all child terms of ‘CHEBI:24431 – chemical entity’, which is the root of the ChEBI ontology. In the CORAL user interface, if the user is selecting a value for ‘Chemical’, the UI would then only allow autocompletion of the names or definitions of terms under CHEBI:24431. Or if the user uploaded a dataset via a spreadsheet, the validator would accept only terms under CHEBI:24431 as valid values for a ‘Chemical’ variable.

The current implementation does not allow a single microtype to be limited to values from multiple ontologies, so the problem of terms with identical names or definitions is not yet applicable in practice. If such a feature were to be added in the future, we would allow the user to see the term IDs in order to distinguish the similar terms. However, this feature would need to be carefully thought out, because allowing the user to make arbitrary choices between very similar terms could easily result in a violation of the principle of interoperability.

> * Regarding JupyterHub in CORAL, is it possible for the user to execute others' notebooks and check if they get the same results as the original author? Are the users able to access the same datasets used by the original author, or do they need special permission in this case?

Yes, this is a key aspect of reproducible data analysis. Our current implementation has a very simple permissions model: everyone with access to the system can read all data and jupyter notebooks added by other users. A more complex model, such as the KBase permissions model, would need to be added as a future improvement to CORAL (in KBase, any user given permissions to view a notebook, called “narrative”, also receives access to the data used in that notebook). We added an explicit statement to that effect to our description of CORAL Jupyter notebooks on p. 27.

> * The title mentions the 'reproducible data analysis. However, the paper does not mention anything about the reproducibility of data analysis using CORAL. The authors should either provide information on 'reproducible data analysis using CORAL' or remove the phrase from the title or rephrase the title as the paper does not mention anything about it.

Reproducibility is conceptually a subclass of reusability. Our focus in CORAL on baking reusability of datasets into the data model also trivially enables reproducibility, e.g., by re-running a Jupyter notebook. We added an explicit mention of this on p. 27.

> * The paper mentions that CORAL failitates adherance to all four of the FAIR principles. However, the paper does not mention anything about Findability and Accessability? Are datasets in CORAL provided with a persistent identifier? Which information in CORAL is provided with a persistent identifier? Please provide more information on this aspect.

We have clarified the REST API section on p. 25 to make this connection more clear. Datasets do have persistent identifiers, which are searchable and retrievable through the remote data access API. In addition to the discussion of this feature on p. 25, we have added entirely new sections to the Supplementary Information to document both our Python and REST APIs.

> Minor Comment:

> Page 4: Space between Reusability and the citation [1,4].

Thank you – we have fixed the error.

Close
