# Supplementary material for: CORAL: A framework for rigorous self-validated data modeling and integrative, reproducible data analysis
Source: Gigascience. 2022 Oct 17;11:giac089. doi: 10.1093/gigascience/giac089 (PMC9575582; doi:10.1093/gigascience/giac089)
Supplement: giac089_Response_to_Reviewer_Comments_Revision_1 [file giac089_response_to_reviewer_comments_revision_1.pdf]

Author's Response To Reviewer Comments

Close

Thank you again for the opportunity to provide this revised manuscript.

We thank the reviewers for careful review of both the original manuscript and our first revision. All changes relative to our original manuscript were tracked using “track changes.” We hope we have addressed all the concerns, as detailed below:

> Reviewer #1: We thank the authors for their efforts to answer the reviewers' questions.

> Outstanding comments:

> 1/. Renaming Results Section to Findings section does not seem possible as this is part of the Giga Science article layout.

This section appears to be appropriate for a Technical Note:

https://academic.oup.com/gigascience/pages/technical_note#Preparing%20main%20manuscript%20text

2/. Cite, in the introduction or general background work about Common Data Elements (CDEs) and ISO11179 specifications, and comment how "contextons" and "microtypes" build on these notions or how they differ.

CDEs are very similar to our dataset-level contextons, and we have added the following text to compare them. The relevant section now reads:

However, a common file format alone is not sufficient to ensure adherence to the FAIR principles of Interoperability and Reusability: in addition to a standard file format, all data, dimensions, and units in these multidimensional arrays must also be formally and rigorously documented. Current state of the art tools such as the CEDAR Workbench [13] greatly facilitate formal documentation of metadata describing entire datasets, but do not readily allow documentation of components such as dimensions and variables. Likewise, Common Data Element (CDE) standards as specified in ISO 111179 [14] have been in use at NIH for over a decade [15], but are not applicable to internal components of data structures.

Later, in discussing dataset-level contextons, we state:

Note that contextons that provide context for the entire matrix are similar to Common Data Elements (CDEs); therefore, microtypes that are compatible with relevant community standards should be included in the set of microtypes defined by the administrators of a CORAL deployment.

Adding rigorous context to all aspects of multidimensional data structures (e.g., the dimension axes of N-dimensional arrays, as well as the data values themselves) is a key aspect of the CORAL data model that was not the focus of CDEs.

3/. REST-API: nice addition to the manuscript. One additional clarifications: is the API compliant with the openAPI/SmartAPI *https://www.openapis.org/* specifications. If the case, it would be another possible point to support FAIR.

OpenAPIs looks like a very useful tool, which we’ll certainly consider using in the next generation of our software, when we re-implement our prototype Python code. As reviewers can see from our current REST API, it’s fairly ad hoc and also only callable by logged in users and authorized remote data access API clients that possess an access token.

A future rewrite of the software that is designed to be accessed by both internal users of an organization as well as the general public (for datasets that have been designated as public) would benefit greatly from a more well-designed and publicly documented API.

4/. Supplementary Material: a lot of material there could have actually be used in the manuscript, eg. figure of the user interface could be used in-lieu of somewhat repetitive segments of figures 4-5-6, simply to break the possible monotony of the pictorial work.

Figures 4, 5, and 6 are essential to understanding the CORAL data model, which we believe is the key innovation in this manuscript. Figure 6 is maybe less critical, and might be moved to the supplementary material if the editors require shortening the main manuscript. We added additional text to the Fig 6 caption to help readers understand when each type of contexton is used:

For example, the data values in an N-dimensional array are N-dimensional contextons, while the values of variables on each dimension axis are 1-dimensional contextons with the same length as each dimension. Single-value contextons are used to provide context for an entire dataset, and as modifiers in building compound contextons.

The screenshots of the user interface from our prototype implementation are of more interest to readers interested in the practical aspects of CORAL, so we believe they would be less critical to include in the main manuscript.

Close
