# Supplementary material for: CORAL: A framework for rigorous self-validated data modeling and integrative, reproducible data analysis
Source: Gigascience. 2022 Oct 17;11:giac089. doi: 10.1093/gigascience/giac089 (PMC9575582; doi:10.1093/gigascience/giac089)
Supplement: giac089_Response_to_Reviewer_Comments_Revision_2 [file giac089_response_to_reviewer_comments_revision_2.pdf]

Author's Response To Reviewer Comments

Close

Thank you - we have made all the requested formatting revisions.

Close
