# Supplementary material for: CORAL: A framework for rigorous self-validated data modeling and integrative, reproducible data analysis
Source: Gigascience. 2022 Oct 17;11:giac089. doi: 10.1093/gigascience/giac089 (PMC9575582; doi:10.1093/gigascience/giac089)
Supplement: giac089_Reviewer_1_Report_Original_Submission — Philippe Rocca-Serra -- 12/8/2021 Reviewed [file giac089_reviewer_1_report_original_submission.pdf]

Reviewer Report

Title: CORAL: A framework for rigorous self-validated data modeling and integrative, reproducible data analysis

Version: Original Submission **Date:** 12/8/2021

Reviewer name: Philippe Rocca-Serra

Reviewer Comments to Author:

We thank the authors for their manuscript.
While the manuscript is well written, I have 2 main comments, one about the manuscript structure and the other about the approach and subsequent claims:
1 manuscript structure: I was puzzled by the Methods section, which seems to mainly contain information that would fall under 'Results'. Under "Methods", I was expecting to find information about software engineering practice, user testing and methodology used to assess FAIRness levels and ontology management libraries / components used, as well as information about the language and data standards used for metadata tracking (e.g. W3C prov-o for provenance information, RDF, JSON, JSON-LD in case those had been used.
Instead, the section contains a long list of components which are specific to CORAL. Can the authors clarify why it is so?
2. the approach and claims:
The authors describe a compositional approach to matrix definitions, relying on "static core types' and 'dynamic type' to define data bricks , why may be reused and shared.
Key elements (Data Category, Dimension Type and Unit) must be tagged with an ontology term. Does Coral come with preloaded/default resources/namespaces for those?Data Category, Dimension Type and Unit must be tagged with an ontology term.
A number of projects have explored these approaches (e.g. CEDAR for metadata description) and ran into the combinatorial explosion of entities. It would seem appropriate for the authors to include a section detailing how to CORAL system help with the status quo?
In keeping with this topic, a number of questions can be raised:
If 'data category' must be set from terms coming from an ontology the selection of which is down to system administrator/ data stewards, how does this help interoperability in the absence of community guidelines or queryable registry?
- Does Coral come with preloaded/default resources/namespaces for those?
- Will Coral provide 'community annotation profiles'?
- If so, what provisions are made to account for versioning of ontological resources?
a number of approaches exist json schema + ajv validator, rdf metadata profiles (shacl + shex expression for validation) . These are not cited or mentioned in the manuscript. Have these been explored in the CORAL work and how they could interface with the approach of microtype definitions? Also, a project such as Biolink.LinkML has made interesting developments, even if at possibly a different level of metadata profile definition, its anchoring in the bio/omics data context could warrant mentionning.

-page 9: "In practice, only a small fraction (~10%) of data types need to be statically defined. " How did the authors come to this estimate? what was the method used?
-page 10: the authors introduce the notion of "data brick", on top of "microtypes" and "contextons", but this appears nowhere in any of the figures, except in Figure 5 where microtype fields are denoted "brick name", "brick ID" when describing an N-dimensional array.
providing a figure more clearly detailing the relationship between these concepts would help the targeted audience.
- Are brick ID resolvable (over https)?
Terms of use and license of microtypes, contextions and brick: providing a example of how to do this would be a nice addition
Figures:
5 figures are devoted to the description of the paradigm. I would recommend condensing those into a synthetic one and use the freed real estate to show case other functionalities (wizard, search interface, visualization) which characterise CORAL.

Level of Interest

Please indicate how interesting you found the manuscript: Choose an item.

Quality of Written English

Please indicate the quality of language in the manuscript: Choose an item.

Declaration of Competing Interests

Please complete a declaration of competing interests, considering the following questions:

- Have you in the past five years received reimbursements, fees, funding, or salary from an organisation that may in any way gain or lose financially from the publication of this manuscript, either now or in the future?
- Do you hold any stocks or shares in an organisation that may in any way gain or lose financially from the publication of this manuscript, either now or in the future?
- Do you hold or are you currently applying for any patents relating to the content of the manuscript?
- Have you received reimbursements, fees, funding, or salary from an organization that holds or has applied for patents relating to the content of the manuscript?
- Do you have any other financial competing interests?
- Do you have any non-financial competing interests in relation to this paper?

If you can answer no to all of the above, write 'I declare that I have no competing interests' below. If your reply is yes to any, please give details below.

I declare that I have no competing interests

I agree to the open peer review policy of the journal. I understand that my name will be included on my report to the authors and, if the manuscript is accepted for publication, my named report including any attachments I upload will be posted on the website along with the authors' responses. I agree for my report to be made available under an Open Access Creative Commons CC-BY license (http://creativecommons.org/licenses/by/4.0/). I understand that any comments which I do not wish to be included in my named report can be included as confidential comments to the editors, which will not be published.

Choose an item.

To further support our reviewers, we have joined with Publons, where you can gain additional credit to further highlight your hard work (see: https://publons.com/journal/530/gigascience). On publication of this paper, your review will be automatically added to Publons, you can then choose whether or not to claim your Publons credit. I understand this statement.

Yes Choose an item.
