# Supplementary material for: CORAL: A framework for rigorous self-validated data modeling and integrative, reproducible data analysis
Source: Gigascience. 2022 Oct 17;11:giac089. doi: 10.1093/gigascience/giac089 (PMC9575582; doi:10.1093/gigascience/giac089)
Supplement: giac089_Reviewer_1_Report_Revision_1 — Philippe Rocca-Serra -- 5/6/2022 Reviewed [file giac089_reviewer_1_report_revision_1.pdf]

Reviewer Report

Title: CORAL: A framework for rigorous self-validated data modeling and integrative, reproducible data analysis

Version: Revision 1 **Date:** 5/6/2022

Reviewer name: Philippe Rocca-Serra

Reviewer Comments to Author:

We thank the authors for their efforts to answer the reviewers' questions.
Outstanding comments:
1/. Renaming Results Section to Findings section does not seem possible as this is part of the Giga Science article layout.
2/. Cite, in the introduction or general background work about Common Data Elements (CDEs) and ISO11179 specifications, and comment how "contextons" and "microtypes" build on these notions or how they differ.
3/. REST-API: nice addition to the manuscript. One additional clarifications: is the API compliant with the openAPI/SmartAPI *https://www.openapis.org/* specifications. If the case, it would be another possible point to support FAIR.
4/. Supplementary Material: a lot of material there could have actually be used in the manuscript, eg. figure of the user interface could be used in-lieu of somewhat repetitive segments of figures 4-5-6, simply to break the possible monotony of the pictorial work.

Level of Interest

Please indicate how interesting you found the manuscript: Choose an item.

Quality of Written English

Please indicate the quality of language in the manuscript: Choose an item.

Declaration of Competing Interests

Please complete a declaration of competing interests, considering the following questions:

- Have you in the past five years received reimbursements, fees, funding, or salary from an organisation that may in any way gain or lose financially from the publication of this manuscript, either now or in the future?
- Do you hold any stocks or shares in an organisation that may in any way gain or lose financially from the publication of this manuscript, either now or in the future?
- Do you hold or are you currently applying for any patents relating to the content of the manuscript?

- Have you received reimbursements, fees, funding, or salary from an organization that holds or has applied for patents relating to the content of the manuscript?
- Do you have any other financial competing interests?
- Do you have any non-financial competing interests in relation to this paper?

If you can answer no to all of the above, write 'I declare that I have no competing interests' below. If your reply is yes to any, please give details below.

I declare that I have no competing interests

I agree to the open peer review policy of the journal. I understand that my name will be included on my report to the authors and, if the manuscript is accepted for publication, my named report including any attachments I upload will be posted on the website along with the authors' responses. I agree for my report to be made available under an Open Access Creative Commons CC-BY license (http://creativecommons.org/licenses/by/4.0/). I understand that any comments which I do not wish to be included in my named report can be included as confidential comments to the editors, which will not be published.

Choose an item.

To further support our reviewers, we have joined with Publons, where you can gain additional credit to further highlight your hard work (see: https://publons.com/journal/530/gigascience). On publication of this paper, your review will be automatically added to Publons, you can then choose whether or not to claim your Publons credit. I understand this statement.

Yes Choose an item.
