# Supplementary material for: CORAL: A framework for rigorous self-validated data modeling and integrative, reproducible data analysis
Source: Gigascience. 2022 Oct 17;11:giac089. doi: 10.1093/gigascience/giac089 (PMC9575582; doi:10.1093/gigascience/giac089)
Supplement: giac089_Reviewer_2_Report_Original_Submission — Sheeba Samuel -- 12/12/2021 Reviewed [file giac089_reviewer_2_report_original_submission.pdf]

Reviewer Report

Title: CORAL: A framework for rigorous self-validated data modeling and integrative, reproducible data analysis

Version: Original Submission **Date:** 12/12/2021

Reviewer name: Sheeba Samuel

Reviewer Comments to Author:

The paper introduces a platform called 'Contextual Ontology-based Repository Analysis Library' (CORAL), which provides support of FAIR data principles, with a particular focus on making heterogeneous data interoperable and reusable using an ontology-based approach.
The framework provides users the facility to add new custom datatypes to extensively document the context of all data based on concepts like microtype, contexton, and ontologies. In addition to this main feature, it also provides support for documenting and uploading datasets, search datasets, plotting wizard for visualization, API access, management dashboard, and Jupytet notebook access through JupyterHub.
The authors have looked into a relevant area by developing a platform that supports FAIR principles to users with the additional benefit of having little maintenance effort. The authors have clearly defined the challenges in supporting FAIR principles in platforms, with discussing on the greater difficulty in the Interoperability and Reusability principles. The text is well written.
The authors provide the code for the system with AGPL license. They also provide installation instructions for CORAL, with separate instructions for frontend and backend part.
Please find below the comments and questions to strengthen this paper:
* The related work is limited. The authors point out only two related software. However, there are tools that provide FAIR support, particularly providing support for adding new custom data types for adding metadata using ontologies. One such example is The CEDAR Workbench: An Ontology-Assisted Environment for Authoring Metadata that Describe Scientific Experiments, 2017. The authors should provide more examples and discuss on comparing CORAL with existing tools used by the community.
* On page 6, the authors mention the survey conducted in the ENIGMA project. In addition to providing the project's website, the authors should also directly point the readers to the resources related to the survey, e.g., survey results.
* The authors mention about ontolgies. However, a definition of the term is missing in the article. The definition of an ontology is provided in Knowledge engineering: Principles and methods. Studer, et al. (1998)
* It is not clear how the software was designed and developed? How are the requirements needed to develop this platform collected? The authors need to provide more information on the experiment design.
* On page 8, the authors mention that the designers of microtypes should endeavor to use community standard ontologies wherever possible: How do the designers ensure that? Are there guidelines for the designers? How are these ontologies selected? Which are the ontologies currently selected in CORAL for

this purpose? The authors should provide more explanation on this point as the platform itself is based on an ontology-based approach.
* On page 12: The authors talk about determining the provenance of the data? It would be good first to give a definition of provenance and also to mention, which provenance information is collected in this system?
* On page 14, the authors mention that the microtype names, as well as other ontological terms, are arranged in hierarchies? Generally, in ontologies, each concept has superclasses and subclasses. How is this hierarchy of the ontologies used and visualized to users in CORAL? Do the authors also make use of the reasoning power provided by ontologies in their system? If yes, the authors need to provide that information to these questions.
* On page 15, the authors provide several benefits of the CORAL approach. The information on how the data is stored is currently is missing. Since there is extensive use of ontological terms, is the data stored as Resource Description Framework (RDF)? If yes, please provide more information on this. If not, please specify how the data and the provenance information are stored and queried by the user?
* On page 17, the authors mention about the validator to limit the values for a microtype. For example, 'Chemical' microtype. A 'Chemical' can appear in multiple ontologies. In that case, who selects which ontologies to be shown for a microtype. If multiple ontologies are shown to the user, how can the user select which term to select from multiple ontologies?
* Regarding JupyterHub in CORAL, is it possible for the user to execute others' notebooks and check if they get the same results as the original author? Are the users able to access the same datasets used by the original author, or do they need special permission in this case?
* The title mentions the 'reproducible data analysis. However, the paper does not mention anything about the reproducibility of data analysis using CORAL. The authors should either provide information on 'reproducible data analysis using CORAL' or remove the phrase from the title or rephrase the title as the paper does not mention anything about it.
* The paper mentions that CORAL failitates adherance to all four of the FAIR principles. However, the paper does not mention anything about Findability and Accessability? Are datasets in CORAL provided with a persistent identifier? Which information in CORAL is provided with a persistent identifier? Please provide more information on this aspect.
Minor Comment:
Page 4: Space between Reusability and the citation [1,4].

Level of Interest

Please indicate how interesting you found the manuscript: Choose an item.

Quality of Written English

Please indicate the quality of language in the manuscript: Choose an item.

Declaration of Competing Interests

Please complete a declaration of competing interests, considering the following questions:

- Have you in the past five years received reimbursements, fees, funding, or salary from an organisation that may in any way gain or lose financially from the publication of this manuscript, either now or in the future?
- Do you hold any stocks or shares in an organisation that may in any way gain or lose financially from the publication of this manuscript, either now or in the future?
- Do you hold or are you currently applying for any patents relating to the content of the manuscript?
- Have you received reimbursements, fees, funding, or salary from an organization that holds or has applied for patents relating to the content of the manuscript?
- Do you have any other financial competing interests?
- Do you have any non-financial competing interests in relation to this paper?

If you can answer no to all of the above, write 'I declare that I have no competing interests' below. If your reply is yes to any, please give details below.

I declare that I have no competing interests

I agree to the open peer review policy of the journal. I understand that my name will be included on my report to the authors and, if the manuscript is accepted for publication, my named report including any attachments I upload will be posted on the website along with the authors' responses. I agree for my report to be made available under an Open Access Creative Commons CC-BY license (http://creativecommons.org/licenses/by/4.0/). I understand that any comments which I do not wish to be included in my named report can be included as confidential comments to the editors, which will not be published.

Choose an item.

To further support our reviewers, we have joined with Publons, where you can gain additional credit to further highlight your hard work (see: https://publons.com/journal/530/gigascience). On publication of this paper, your review will be automatically added to Publons, you can then choose whether or not to claim your Publons credit. I understand this statement.

Yes Choose an item.
